# Network Segregation and Integration Changes in Healthy Aging: Evidence From EEG Subbands During the Visual Short‐Term Memory Binding Task

**DOI:** 10.1111/ejn.70001

**Published:** 2025-02-05

**Authors:** Ezgi Fide, Emre Bora, Görsev Yener

**Affiliations:** ^1^ Department of Psychology, Faculty of Health York University Toronto Ontario Canada; ^2^ Department of Neurosciences, Institute of Health Sciences Dokuz Eylül University Izmir Turkey; ^3^ Faculty of Medicine, Department of Psychiatry Dokuz Eylül University Izmir Turkey; ^4^ Faculty of Medicine, Department of Neurology Dokuz Eylül University Izmir Turkey; ^5^ Izmir International Biomedicine and Genome Institute Dokuz Eylül University Izmir Turkey

**Keywords:** aging, graph theory analysis, integration, segregation, SW, working memory

## Abstract

Working memory, which tends to be the most vulnerable cognitive domain to aging, is thought to depend on a functional brain network for efficient communication. The dynamic communication within this network is represented by segregation and integration. This study aimed to investigate healthy aging by examining age effect on outcomes of graph theory analysis during the visual short‐term memory binding (VSTMB) task. VSTMB tasks rely on the integration of visual features and are less sensitive to semantic and verbal strategies. Effects of age on neuropsychological test scores, along with the EEG graph‐theoretical integration, segregation and global organization metrics in frequencies from delta to gamma band were investigated. Neuropsychological assessment showed low sensitivity as a measure of age‐related changes. EEG results indicated that network architecture changed more effectively during middle age, while this effectiveness appears to vanish or show compensatory mechanisms in the elderly. These differences were further found to be related to cognitive domain scores. This study is the first to demonstrate differences in working memory network architecture across a broad age range.

## Graphical abstract

We showed that network architecture changes during healthy aging are more effective in middle‐aged individuals, while this effectiveness appears to vanish or show compensatory mechanisms in elderly people. These changes were related to cognitive domain scores. This study is the first to demonstrate differences in working memory network architecture across a broad age range.

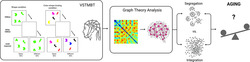

**Abbreviations**
bc
: betweenness centrality
BDI: Beck Depression Inventory
CC: clustering coefficient
CPL: characteristic path length
EEG: electroencephalography
EOGs: electrooculograms
GAM: generalized additive models
HERMES: Herramientas de Medida de la Sincronización Tools for the Assessment of Synchronization
MMSE: Mini‐Mental State Examination
SW: small world
TMT: Trail Making Test
VSTMB: visual short‐term memory binding
WAIS: Wechsler Adult Intelligence Scale
WMS‐R: Wechsler Memory Scale‐Revised
wPLI: weighted phase lag index

## 1 Introduction

Brain structures and cognitive processing change dynamically throughout an individual's life (Salthouse 2010a, 2010b; Fjell and Walhovd 2010). Healthy aging depends on the preservation of cognitive and functional abilities and the capacity to resist various environmental, behavioural and genetic factors that put these abilities at risk (Cohen, Marsiske, and Smith 2019). Among cognitive domains, working memory, attention and executive functions stand out as particularly vulnerable to aging (Rose et al. 2009; Johnson, Logie, and Brockmole 2010; Basak and Verhaeghen 2011; Bopp and Verhaeghen 2020). The importance of working memory relies on its pivotal role in supporting many cognitive functions, and its capacity is central to activities of daily living (Baddeley 1986; Unsworth et al. 2014; Borella et al. 2007; D'Esposito and Postle 2015; Gutchess and Boduroglu 2019). Working memory performance is associated with the interaction between attention, short‐term retention and manipulation of information by the coactivation of many brain regions (Eriksson et al. 2015).

Working memory is thought to rely on a functional brain network for efficient communication and maintenance of modularity (Iordan et al. 2021). These information‐processing patterns within the brain dynamically change and are represented by two functional states of the brain network: (1) segregation, which is the independence of processing in specialized subsystems, and (2) integration, which is the efficiency of global information transmission or the ability to combine distributed information from various subsystems (Sporns 2013; Bassett and Bullmore 2017; Zippo et al. 2018; Shine 2019; Farahani, Karwowski, and Lighthall 2019; Wang et al. 2021). It is suggested that higher segregation is associated with simple motor tasks, while higher integration underlies performance in tasks with higher cognitive load (Braun et al. 2015; Chen and Deem 2015; Cohen and D'Esposito 2016; Shine et al. 2016; Kolskår et al. 2018). Stronger integration is associated with higher general cognitive ability, whereas greater network segregation indicates better crystallized intelligence and supports information‐processing speed (Wang et al. 2021). Small world (SW), on the other hand, serves as an indicator of the homeostatic balance between the randomness and order within the dynamic system organization where separation and integration reach an optimal level (Rubinov and Sporns 2010; Cao et al. 2016; Hong et al. 2016; Hou et al. 2018).

Electroencephalography (EEG) stands out among other methods due to its temporal resolution, revealing oscillations at various frequencies, that represent synchronized activity within the neuronal network. EEG is important for analysing how complex networks will likely function (Mheich, Wendling, and Hassan 2020). It is worth noting that network properties can undergo changes during healthy aging. Studies have demonstrated that connectivity in the theta and alpha frequency bands decreased (Hou et al. 2018), while beta connectivity increased with age (Wang et al. 2017; Hou et al. 2018). Similarly, stronger beta phase synchronization has been reported in elderly group compared to young (Hong et al. 2016; Wang et al. 2017). An increased beta response has also been observed during the memory load task (Zarahn et al. 2007). Additionally, lower cognitive reserve is associated with higher functional connectivity (López et al. 2014). Earlier studies in similar research interest have shown both visual short‐term memory capacity, known to decrease with age, and task load can be reflected in central EEG activity, and this activity also undergoes age‐related changes (Sander, Werkle‐Bergner, and Lindenberger 2011; Wiegand et al. 2014, 2018).

Despite the well‐documented alterations in brain activities and functional connectivity during healthy aging, it is important to note that aging does not solely affect activities within specific functional networks; it also disrupts communication between different functional networks (Geerligs, Maurits, Renken, and Lorist 2014; Geerligs, Saliasi, et al. 2014; Hong et al. 2016; Javaid, Kumarnsit, and Chatpun 2022). In this context, graph theory analysis is an ideal tool for examining changes in both global connectivity and local networks, allowing for correlation of neural changes with cognitive functions (Meunier, Stamatakis, and Tyler 2014). Graph theory analysis can indicate features of this communication by providing valuable insights into the presence of direct integration of brain areas or functionally segregated clusters of brain regions (Hinault et al. 2021). Such analysis is also essential for investigating network resilience and robustness (Arul et al. 2023).

A thorough evaluation of the brain network enables a rigorous assessment of its structural stability by examining network efficiency and modularity, as well as the importance of each node through an assessment of hubness, betweenness centrality (bc) and clustering coefficient (CC) (Ma et al. 2021; Zamani Esfahlani et al. 2021; Lee et al. 2023; Mamat et al. 2024). All these properties uncover different aspects of the brain network and enable the assessment of brain network functionality (Pievani et al. 2014). Notably, high CC and sparse characteristic path length (CPL) in networks can minimize metabolic cost. This optimization is crucial for achieving optimal temporal resolution, especially for effective connectivity analysis. Additionally, bc, defined as a node's capacity to relay information along the shortest paths between nodes, plays a significant role in this process (Xu et al. 2023).

Previous studies conducting graph theory analysis have mostly investigated aging effect during rest condition. These studies showed that while resting state networks were more random in the young and elderly group, they are more structured in the middle‐aged group, and SW values are lower in the elderly group (Gaál et al. 2010; Smit et al. 2010). Betzel and Bassett (2017) reported a decline in SW with aging, reflecting reduced efficiency and integration in brain networks. In a study conducted with 113 healthy individuals, it was stated that the alpha CPL gradually shorten from young to the elderly, while in the delta and theta bands, CPL were longest in the elderly group (Vecchio et al. 2014). Recently, it has been shown that functional network segregation declines during aging, with significant deterioration beginning in the late fifties, and this diminished segregation has negative impacts on general cognitive functions (Pedersen et al. 2021).

There are also a few studies that have delved into the working memory networks in the context of aging. Geerligs, Saliasi, et al. (2014) found that older adults exhibit less efficient network organization compared to young individuals, highlighting the impact of aging on brain network integration and segregation. A recent study showed pronounced differences between middle‐aged and elderly groups, demonstrating greater CC, CPL, global efficiency, local efficiency and node strength in the middle‐aged group (Javaid, Kumarnsit, and Chatpun 2022). Hong et al. (2016) examined the effects of aging on brain networks using the go/no‐go task. They found no difference in SW properties; however, node distribution in the theta and beta bands was more prominent in older adults than in young adults. Hou et al. (2018), employing the *N*‐back task, revealed increased CC of beta‐band connectivity in young adults compared to older adults. More recently, focusing on working memory capacity, authors showed alterations in CC, SW, global efficiency and CPL metrics across young and elderly groups (Xu et al. 2023). Their findings suggest a decline in network connectivity and less differentiated or specific functional networks in the elderly, as evidenced by lower CC, while the proportion of long‐distance connections decreased with aging, reflected in lower CPL.

However, measuring working memory alone can pose challenges due to its verbal nature. This verbal aspect may predispose individuals to semantic interference and reliance on cognitive reserves—the ability to find alternative ways of performing a task to compensate for deficiencies (Stern 2009). In contrast to the previously mentioned working memory tasks, visual short‐term memory binding (VSTMB) tasks require the integration of visual features and are less influenced by semantic and verbal strategies (Pavisic, Suarez‐Gonzalez, and Pertzov 2020). The VSTMB supports the temporary retention of associations or features (e.g., colours and shapes) as integrated complex objects (Luck and Vogel 1997; Treisman and Zhang 2006). Accurately constructing memory representations of complex objects requires effective connectivity among brain regions involved in feature processing (Lafer‐Sousa and Conway 2013).

The VSTMB tasks are also effective in assessing functionality in daily life and can accurately detect pathological aging processes. Previous studies reported that VSTMB tasks are quite successful in detecting Alzheimer's disease (Parra, Abrahams, Logie, Méndez, et al. 2010; Parra, Abrahams, Logie, and Della Sala 2010; Parra et al. 2017; Della Sala et al. 2018), and they even have been proposed as a marker for Alzheimer's disease (Cecchini et al. 2021); but they have limited success in detecting changes associated with healthy aging (Parra, Abrahams, Logie, and Sala 2009; Parra, Abrahams, Fabi, et al. 2009; Rhodes, Parra, and Logie 2016; Brown et al. 2017). A study of more than 55,000 participants reported a significant (but weak) age‐related deterioration in binding (Brockmole and Logie 2013). Compared to young adults, older adults exhibit a marked deficit in memory related to binding features. Yet, assessment of age differences in change detection performance by manipulating attentional load, coding time or the holding interval showed intact ability of older adults to detect binding changes (Brockmole et al. 2008; Parra, Abrahams, Logie, and Sala 2009; Rhodes, Parra, and Logie 2016). Nevertheless, using the same paradigm, Brown and Brockmole (2010) found that visual binding disruption due to aging can indeed accompany the healthy aging process.

These findings suggest that the VSTMB task can be sensitive to aging and capable of detecting age‐related changes, even though studies on healthy aging have yielded inconsistent results (Parra, Abrahams, Logie, and Sala 2009; Parra, Abrahams, Fabi, et al. 2009; Brown and Brockmole 2010; Brockmole and Logie 2013; Rhodes, Parra, and Logie 2016; Brown et al. 2017). Interestingly, previous studies have often compared differences between young and old groups, or they have included middle‐aged and elderly individuals in the same group when comparing them to young adults. To date, no previous study has investigated changes between young and middle‐aged groups' working memory‐related brain networks, despite the well‐established onset of cognitive declines in middle age. It has been suggested that examining feature binding in aging will help prepare screening tools to distinguish pathological aging (Holcomb, Tagliabue, and Mazza 2022). It can also help implement interventions and preventive measures against healthy aging.

Given the well‐documented effects of healthy aging on working memory and visual memory, measuring dynamic brain networks during the VSTMB task promises to provide more comprehensive findings. In this context, network analysis methods can guide the determination of the source of the problem. Thus, this study aimed to investigate healthy aging by analysing its effect with regression approach using graph theory analysis to assess segregation, integration and SW during the VSTMB task. We hypothesize that (1) age will affect segregation and integration measures, leading to reduced segregation and integration with increasing age (Sander, Lindenberger, and Werkle‐Bergner 2012; Geerligs, Saliasi, et al. 2014); (2) global organization characteristics (i.e., SW) will decrease with healthy aging, indicating less efficient network organization in older adults (Cao et al. 2016; Xu et al. 2023); (3) conditions of the VSTMB task will alter network characteristics, with more complex condition requiring greater network adaptability (Palva et al. 2010; Alavash, Thiel, and Gießing 2016); and (4) an exploratory correlation analysis between graph metrics that show an effect of age and neuropsychological test scores will be conducted to further explore possible relations.

## 2 Materials and Methods

**FIGURE 1:**
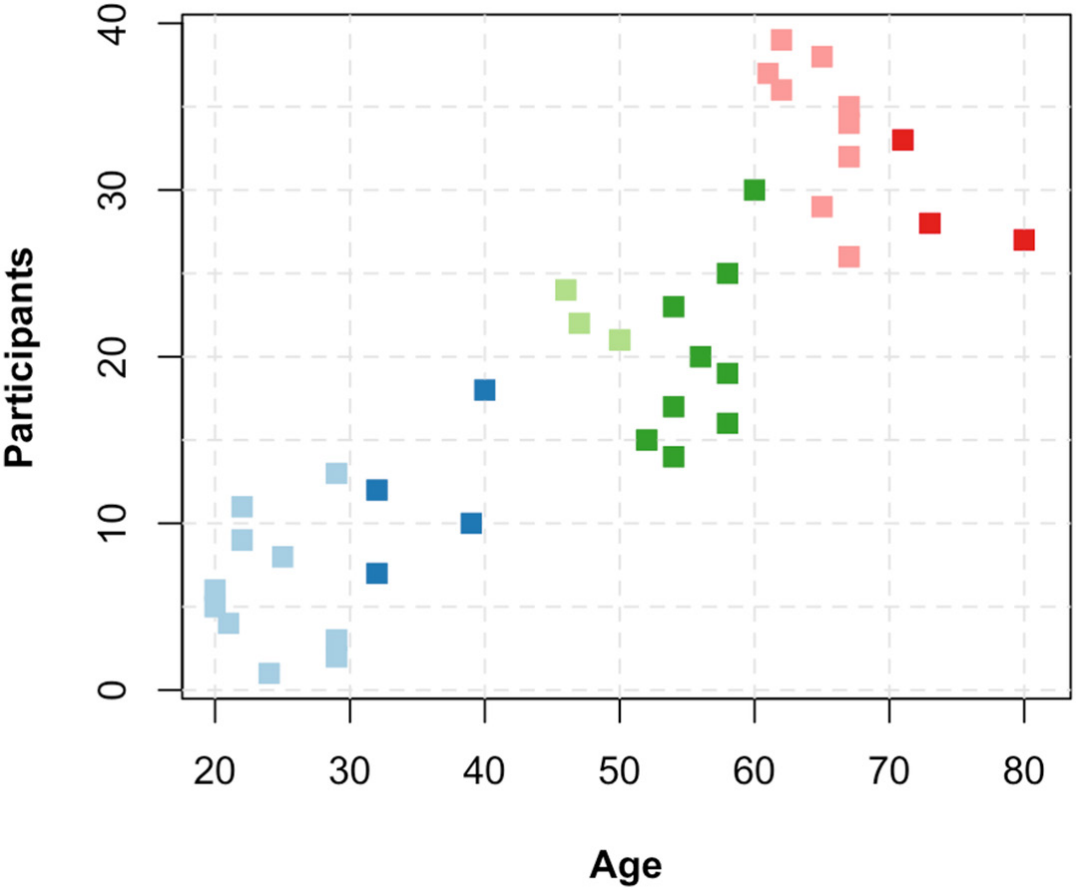
Participants' age distribution.

Data for this cross‐sectional study were collected from a sample of 40 individuals aged 20–81 years (Figure 1), all of whom had no neurological or psychiatric history and no family history of dementia. Individuals who volunteered to participate in the study were reached through various community platforms. Comprehensive neuropsychological tests, including the depression scale, general cognitive assessment, episodic and visual memory, attention, executive functions, language skills and visuospatial skills assessments, were performed to evaluate the participants' cognitive status. We obtained informed consent for the study from all participants prior to the tests. Only participants scoring within the norm values (−1 standard deviation) were included in the EEG analyses.

The exclusion criteria for all participants were (1) Beck Depression Inventory (BDI; Beck et al. 1961; Tegin 1980) score above 17; (2) Mini‐Mental State Examination (MMSE; Güngen et al. 2002) score below 27; (3) presence of B12 deficiency and iron anaemia, substance abuse or use of drugs (benzodiazepines, etc.) that actively affect the central nervous system; and (4) history of neurological conditions such as stroke, head trauma or epilepsy. One participant was excluded from the study due to test scores falling outside the norm. Therefore, the final sample consisted of 39 participants, and EEG recordings were performed. This study confirmed the principles of the Declaration of Helsinki and was approved by the local ethical committee of the Dokuz Eylül University. The demographic characteristics of the participants are presented in Table 1.

**TABLE 1:** Demographic characteristics of the participants.

	Participants	Intercept estimate	Intercept SE	Intercept *t* value	Intercept *p* value	EDF	Ref. *df*	*F* value	*p* value
*n*	39	—	—	—	—	—	—	—	—
Age	49.21 (18.44)	—	—	—	—	—	—	—	—
Education	16.15 (2.88)	—	—	—	—	—	—	—	—
Sex (F/M)	24/15	—	—	—	—	—	—	—	—
Handedness (R/L/Both)	36/2/1	—	—	—	—	—	—	—	—
MMSE	29.41(1.04)	29.41	0.13	230.71	< 0.001	3.24	4.01	7.12	**< 0.001**
BDI	6.95 (5.02)	6.76	0.84	8.10	< 0.001	2.41	3.00	2.18	0.108
Epoch numbers									
Shape	38.05 (6.24)	—	—	—	—	—	—	—	—
Colour‐shape binding	39.28 (6.60)	—	—	—	—	—	—	—	—

*Note:* The data are presented as means (standard deviations). Bold values denote statistical significance at *p* < 0.05.

Abbreviations: EDF = effective degrees of freedom; F = female; L = left; M = male; R = right; Ref. *df* = reference degrees of freedom; SE = standard error.

### 2.1 Neuropsychological Measures

A comprehensive neuropsychological test battery is applied to all participants to obtain scores for the six cognitive domains, including *episodic memory* (Oktem Verbal Memory Processes Test [Tanör 2011]), *visual memory* (WMS‐R visual reproduction subtest [Wechsler 1987]), *attention* (WMS‐R digit span tests [Wechsler 1987], Trail Making Test [TMT] Parts A and B [Cangöz, Karakoc, and Selekler 2009], WMS‐III mental control tests [Wechsler 1997a]), *executive functions* (WAIS similarities and proverbs [Wechsler 1997b], Wisconsin card sorting test [Spreen and Strauss 1998], Stroop test interference time [Emek Savaş et al. 2020], TMT B‐A time scores), *language skills* (fluency tests [Tumaç 1997] and Boston naming test [Kaplan, Goodglass, and Weintraub 2001]) and *visuospatial abilities* (clock‐drawing test [Cangöz, Karakoç, and Selekler 2006], Benton Judgement of Line Orientation [Benton, Varney, and Hamsher 1978], simple copying test [Wechsler 1987]).

### 2.2 EEG Recording, Paradigm and Preprocessing

EEG recordings were conducted during morning hours (approximately between 09:00 and 10:00 am) and each session lasted ≈ 15 min. Participants were seated in an isolated room during the recordings. EEG signals were captured using 30 Ag/AgCL electrodes placed on an elastic cap (EasyCap; Brain Products GmbH) based on the international 10–20 system, with reference to linked earlobe electrodes (A1 + A2). Electrooculograms (EOGs) were recorded from the upper medial and lateral orbital areas of the eye. The EEG and EOG signals were digitized at a sampling frequency of 500 Hz and amplified using a Brain Amp 32‐channel DC system machine equipped with a 0.1‐ to 70‐Hz band‐pass filter. The impedance values of all electrodes were maintained below 10 kΩ.

**FIGURE 2:**
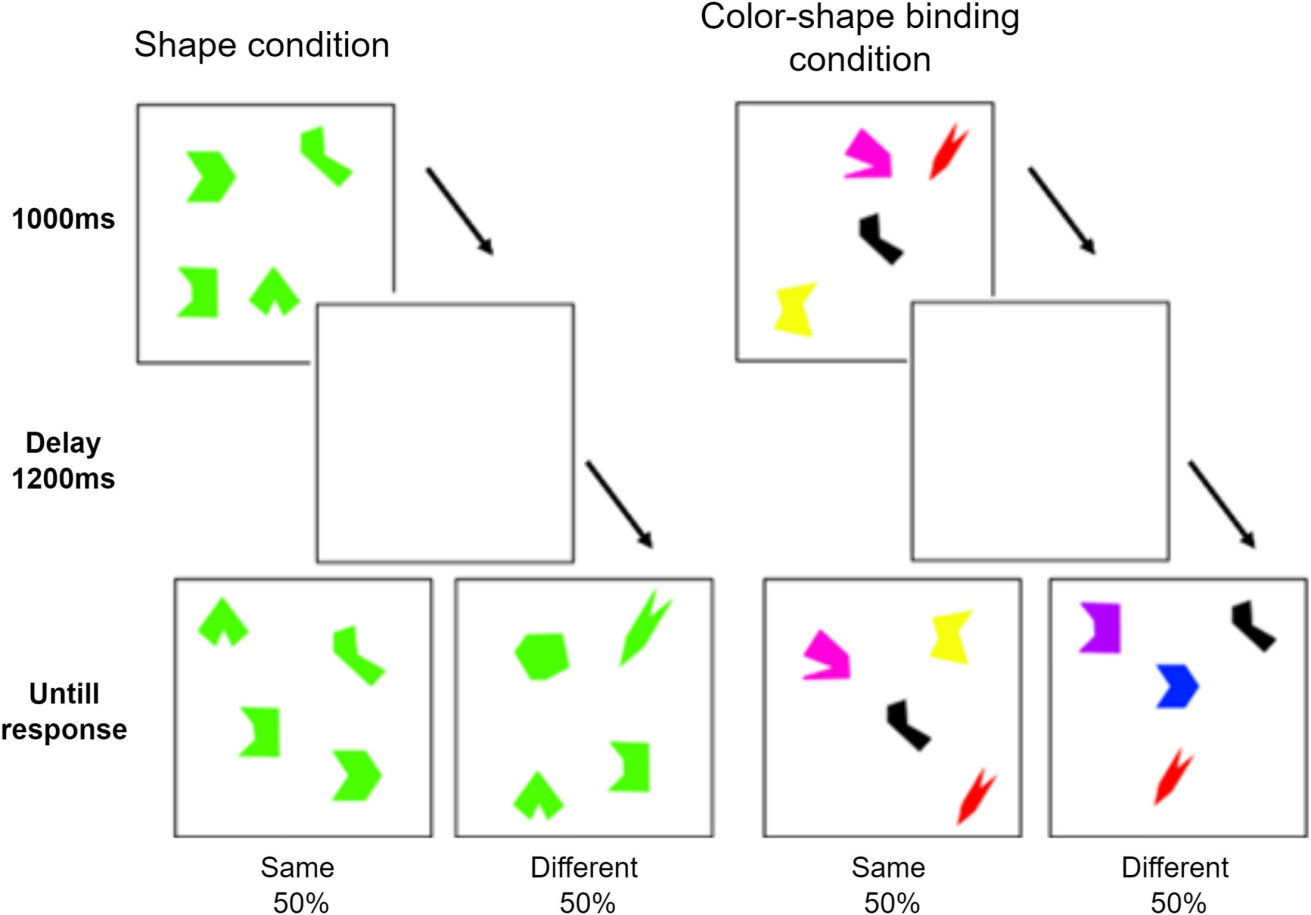
The VSTMB task paradigm schema.

During the EEG recordings, the participants were instructed to complete the VSTMBT paradigm (Parra, Abrahams, Logie, Méndez, et al. 2010) (Figure 2), while their EEG activities and responses were recorded. The VSTMBT required participants to remember four visual arrays consisting of either different shapes in the same colour (shape condition) or different coloured shapes (colour‐shape binding condition), each presented for 1000 ms. After a 1200‐ms delay, a test display appeared, presenting the same or different objects, now in new random locations. The test screen displayed the same or different objects randomly. Participants were then required to decide whether the objects in the test display were the same or different compared to the initial display. The probability of the test display being the same or different was 50% and was randomly determined. Participants indicated their decision by pressing the ‘E’ key for ‘same’ and the ‘I’ key for ‘different’. The maintenance screen remained paused until the participant responded. EEG recording was completed after 96 stimuli were presented, including 48 for encoding and testing in each condition. Conditions were counterbalanced across participants.

Offline analysis was performed using the Brain Vision Analyzer 2.2 program. First, the sampling rate was reduced to 256 Hz, and a 50‐Hz notch filter was applied to the raw data to eliminate the city grid artefact. To correct muscle and eye movements and identify components containing movements, independent component analysis (ICA) with the Infomax‐Extended (Bell and Sejnowski 1997) method was applied to the continuous EEG data. The data were further segmented into 1000 ms epochs time‐locked to stimulus onset at the test screens. Remaining artefacts, such as mechanical and perspiration, were removed semi‐automatically based on the following criteria: (a) amplitude threshold of ±70 μV, (b) maximum allowed voltage step of 50 μV/ms, (c) maximum allowed difference in a 200 ms interval of 50 μV and (d) lowest activity in a 100 ms interval of 0.5 μV. For the graph theory analysis, only the test period was examined.

### 2.3 Weighted Phase Lag Index (wPLI)

The challenge of indexing EEG phase synchronization can be listed as the common reference interference, volume‐conduction effect, noise contamination and sample‐size bias (Vinck et al. 2011). To address these challenges, various techniques have been proposed, among which the wPLI has emerged as particularly promising due to its sensitivity in detecting phase synchronization and related changes (Vinck et al. 2011). Previous studies have demonstrated the efficacy of wPLI in capturing synchronized EEG activity (Vinck et al. 2011; Lau et al. 2012; Ortiz et al. 2012; Yoshinaga et al. 2020). Thus, in this study, we employed wPLI analysis to generate connectivity matrices.

**FIGURE 3:**
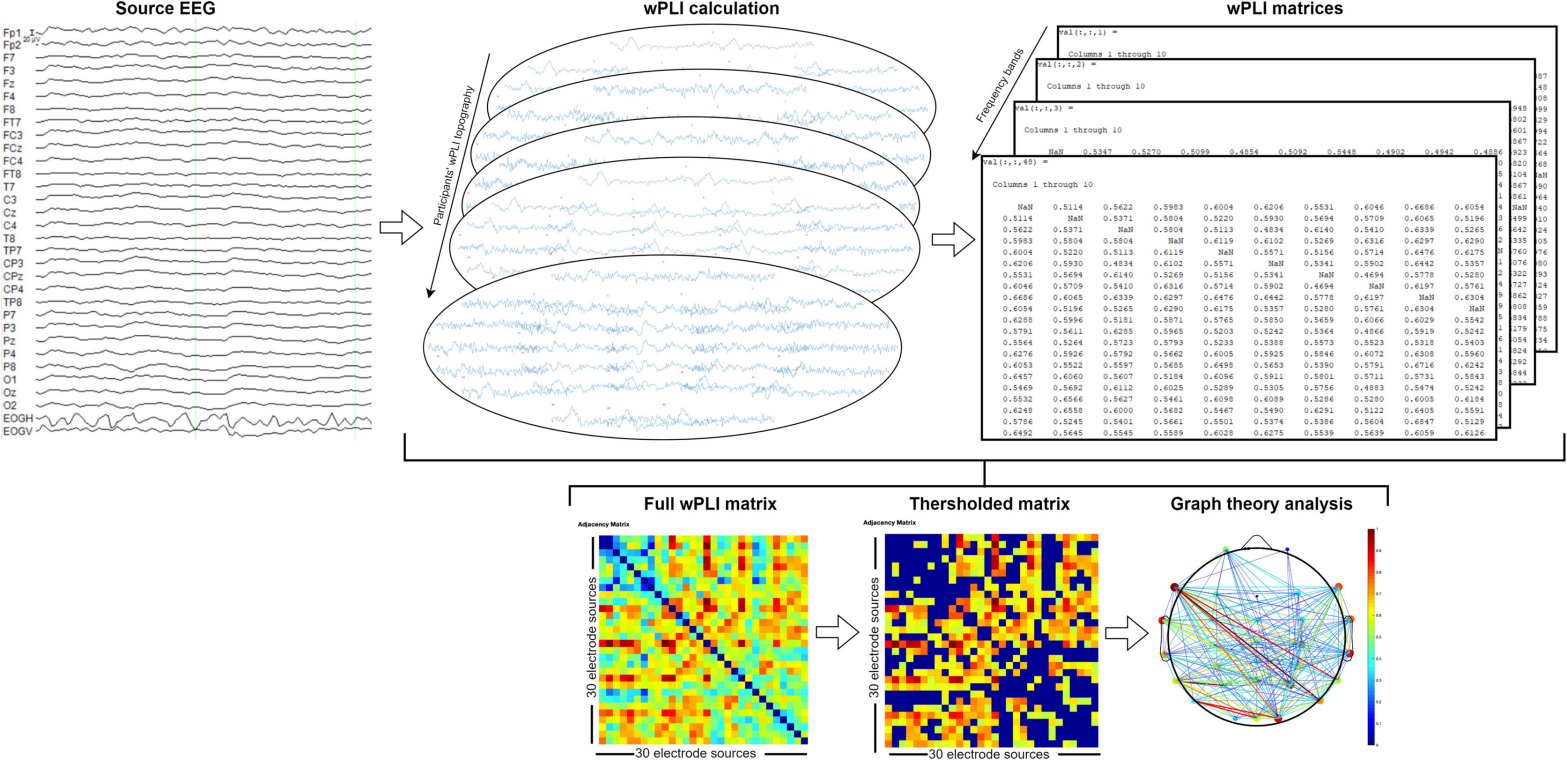
EEG analysis pipeline. Data are preprocessed and divided into stimulus‐locked epochs. For each time and frequency points, a full adjacency matrix containing wPLI estimates is created. Each weighted and undirected matrix is thresholded. The topology of each graph is characterized by graph measures.

The EEG data prepared for analysis were converted into matrices with dimensions *m* (number of electrodes/nodes) × *n* (samples) × *t* (number of epochs) using the EEGLAB program. wPLI analysis was performed using the HERMES program (Niso et al. 2013). The parameters for wPLI were set as follows: central band frequency range of 1–48 Hz with a bandwidth of 1 Hz. This allows for wPLI measurements to be conducted across the delta to the gamma frequency bands, covering the 0.5‐ to 48.5‐Hz range with increment of 1 Hz (Figure 3).

### 2.4 Graph Theory Analysis

Undirected and weighted matrices were generated from HERMES for each person and condition and then imported into EEGNet (Lawhern et al. 2018). A threshold value of 55% was determined based on the CPL and CC values. Graph metrics were calculated for the delta (0.5–3.5 Hz), theta (4–7.5 Hz), alpha (8–13.5 Hz), beta (14–29.5 Hz) and gamma (30–48.5 Hz) frequency bands across the conditions (Figure 3).

To determine the SW values, a 30 × 30 lattice graph was created using MATLAB program. The CPL (*L*) and CC (*C*) values were obtained from the lattice graph, and the *L* (*L*_rand_) and *C* (*C*_rand_) values were computed on this artificial graph. Ratios of the participants' *L* and *C* values to the corresponding *L*_rand_ and *C*_rand_ values were then calculated: $\begin{array}{c} \gamma =\;L/\left(L\;\left(\mathrm{rand}\right)\right)\;\lambda =\;C/\left(C\;\left(\mathrm{rand}\right)\right)\;\\ \mathrm{SW}=\sigma =\;\gamma /\lambda \end{array}$.

### 2.5 Graph Theory Metrics

**FIGURE 4:**
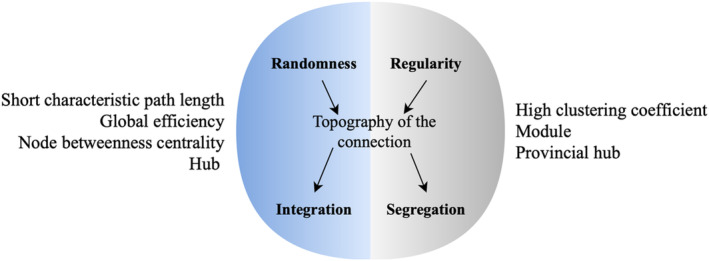
Graph theory analysis metrics that were calculated to define integration and segregation features of the participants' brain networks.

Commonly calculated graph theory metrics for segregation and integration, which were also measured in this study, are presented in Figure 4.

#### 2.5.1 Integration

The most commonly calculated metric within graphs is the CPL, which denotes the number of edges passed when moving from one node to another. A shorter CPL supports efficient parallel information transfer and demonstrates high global integration of connections (Medaglia et al. 2018). Another important metric is global efficiency, which quantifies the information exchange across the entire network and is defined as the inverse of the average path length. It is a measure of ‘how efficiently information flows in a network’ (Latora and Marchiori 2001). The higher the global efficiency values, the faster the information flows, and they are associated with greater integration (Leitgeb et al. 2020). The node bc is equal to the number of shortest paths passing through that node (Javaid, Kumarnsit, and Chatpun 2022). A node with high bc has a great influence on the information flow in the network. A hub is characterized as a node with a high degree or high centrality. Nodes are classified as a hub if their intermodule connectivity scores exceed the defined threshold; otherwise, they are classified as nonhubs (Sporns and Betzel 2016).

#### 2.5.2 Segregation

CC is a key metric for segregation and represents the clustering tendency of the nodes within the network. The large CC indicates that the network consists of highly clustered nodes (Mheich, Wendling, and Hassan 2020). Another segregation metric is the module, which signifies the extent to which a node is densely interconnected compared to others (Meunier, Lambiotte, and Bullmore 2010; Sporns and Betzel 2016). Additionally, a provincial hub is a node that primarily links vertices within a single cluster (Sporns, Honey, and Kötter 2007).

#### 2.5.3 Global Organization

SW is a feature of some networks where most nodes are not adjacent to each other but can be reached by a small number of steps from any given node. SW networks are characterized by greater CC and shorter CPL (Watts and Strogatz 1998). In a real network system, the ratio of CC to the real‐world network should be > 1, while the ratio of CPL to the real‐world network should be ≈ 1 (Maslov and Sneppen 2002; Sporns and Zwi 2004; Achard et al. 2006; Sporns 2007).

### 2.6 Statistical Analysis

To examine the effects of age, we employed generalized additive models (GAM) due to their flexibility in modelling non‐linear relationships between predictors and outcomes. Unlike the generalized linear models, where the linear predictor is *η* = ∑_*j*_ *β*_*j*_ *x*_*j*_ (*η* is the linear predictor, *β*_*j*_ are the coefficients and *x*_*j*_ are the predictor variables), GAM replace these linear terms with smooth functions, resulting in *η* = ∑_*j*_ *f*_*j*_(*x*_*j*_), where *f*_*j*_(*x*_*j*_) represents nonparametric smooth functions of the predictors. This extension allows for more nuanced modelling of complex, non‐linear relationships (McCullagh and Nelder 1989). These smooth functions can be estimated using smoothing splines (Green and Silverman 1994). In GAM with regression splines, *f*_*j*_(*x*_*j*_) is often defined using methods like natural cubic splines or B‐splines, with a predetermined number of knots at specified locations (de Boor 1978). Additionally, unlike linear regression models, GAM require iterative approximations to optimize the estimates (Dominici et al. 2002).

In this study, statistical analyses were performed using the R programming language, specifically utilizing the ‘mgcv’ package (Wood 2017). We used smooth functions of age in years, represented by cubic regression splines. For cognitive outcomes, we used a smooth term of order 10, while for graph‐theoretical outcomes, we used a smooth term of order 12. The smoothness chosen based on model fit criteria.

The relationship between the graph metrics and the BDI, MMSE, cognitive domains and behavioural data of the groups was examined using partial Pearson correlation analysis, with age controlled as a covariate. All analyses were performed by including the whole participants. Correlations that are moderate or above (|*r*| > 0.400; Dancey and Reidy 2007) and remaining statistically significant after Benjamin–Hochberg false discovery rate (Efron 2007) correction were reported.

## 3 Results

### 3.1 Behavioural Data and Neuropsychological Characteristics of Participants

Table 2 represents the results from GAM analysis for behavioural and cognitive outcomes, examining the effect of age. Older participants show decreased response accuracy and increased reaction times. The model indicated a significant effect of age on response accuracy in shape condition (*p* < 0.001), revealing a decline with age, particularly after the age 50. For reaction time, older participants exhibited significantly longer reaction times compared to younger participants, particularly after the age 65 (*p* = 0.006). For binding condition, response accuracy also showed an age effect (*p* = 0.016), with accuracy increasing at younger ages, decreasing in middle age and fluctuating further in later years. The age effect on reaction time was significant (*p* = 0.015), with reaction time increasing particularly sharply after age 70, indicating a substantial slowing of reaction time with age.

**TABLE 2:** Summary of GAM analysis of behavioural data and cognitive domain scores, adjusted for participants' age.

	Intercept estimate	Intercept SE	Intercept *t* value	Intercept *p* value	EDF	Ref. *df*	*F* value	*p* value
Accuracy rate (%)								
Shape condition	73.50	1.48	49.63	< 0.001	4.87	5.87	6.41	**< 0.001**
Colour‐shape binding condition	65.28	1.51	43.19	< 0.001	4.77	5.76	3.19	**0.016**
Reaction time (ms)								
Shape condition	1975.10	110.68	17.85	< 0.001	7.76	8.60	3.46	**0.006**
Colour‐shape binding condition	1970.60	105.15	18.74	< 0.001	6.28	7.38	2.97	**0.015**
Episodic memory	0.00	0.09	0.00	1.00	1.91	2.39	3.09	**0.047**
Visual memory	0.00	0.09	0.00	1.00	5.52	6.56	9.37	**< 0.001**
Attention	−0.03	0.09	−0.29	0.77	3.98	4.87	7.01	**< 0.001**
Executive functions	−0.04	0.08	−0.53	0.60	2.30	2.87	7.16	**0.001**
Language skills	−0.02	0.10	−0.20	0.84	2.89	3.60	9.28	**< 0.001**
Visuospatial abilities	−0.03	0.07	−0.42	0.68	6.03	7.13	13.88	**< 0.001**

*Note:* Bold values denote statistical significance at *p* < 0.05.

Abbreviations: EDF = effective degrees of freedom; Ref. *df* = reference degrees of freedom; SE = standard error.

Visual memory, attention, executive functions, language skills and visuospatial abilities all show significant non‐linear relationships with age, suggesting complex patterns of change. More detailly, the analysis revealed a significant effect of age on episodic memory and attention, with older participants, especially after the age 50, performing worse (*p* = 0.047 and *p* < 0.001, respectively). The visual memory also revealed a significant non‐linear effect of age (*p* < 0.001), suggesting that visual memory worsens with age particularly after age 60.

The model of age effect on executive functions indicated a significant non‐linear relationship (*p* = 0.001), showing that executive function scores remained consistent until middle age, after which they began to decrease. Language skills demonstrated a significant non‐linear age effect (*p* < 0.001), reflecting an increase until age 45, followed by a decline. Visuospatial abilities were significantly affected by age (p < 0.001), with a sharp decline observed after age 70.

### 3.2 Graph Theory Analysis Findings

The model's intercepts were statistically significant across all outcomes (*p* < 0.001), indicating that the baseline levels for each metric were robustly estimated. The effect of age, modelled as a smooth term, exhibited varying degrees of significance depending on the specific outcome and frequency band.

#### 3.2.1 Integration Metrics

**FIGURE 5:**
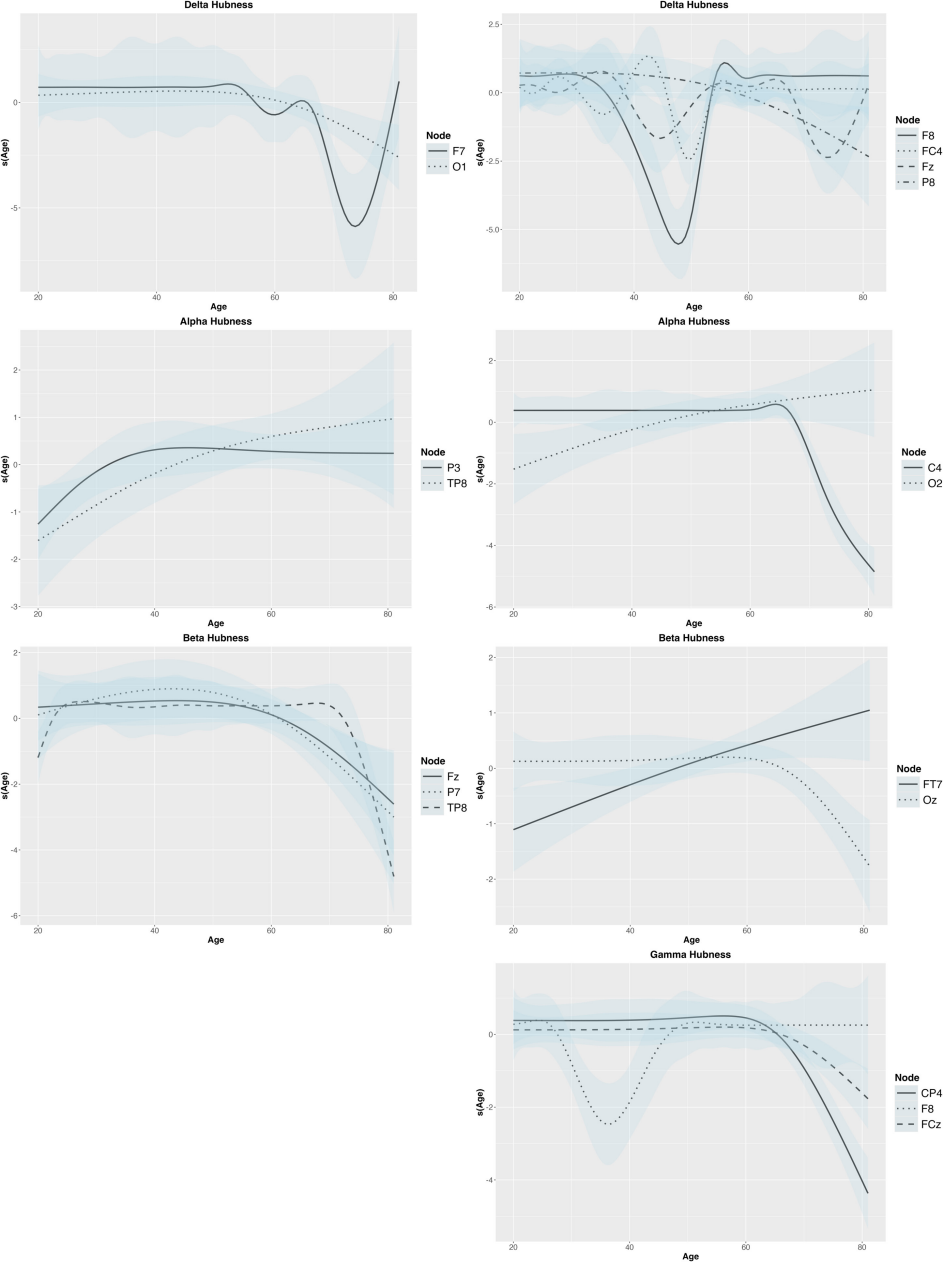
The age effect on hub values showed significant relationships across the nodes. The right side represents the shape condition, and the left side represents the binding condition.

**FIGURE 6:**
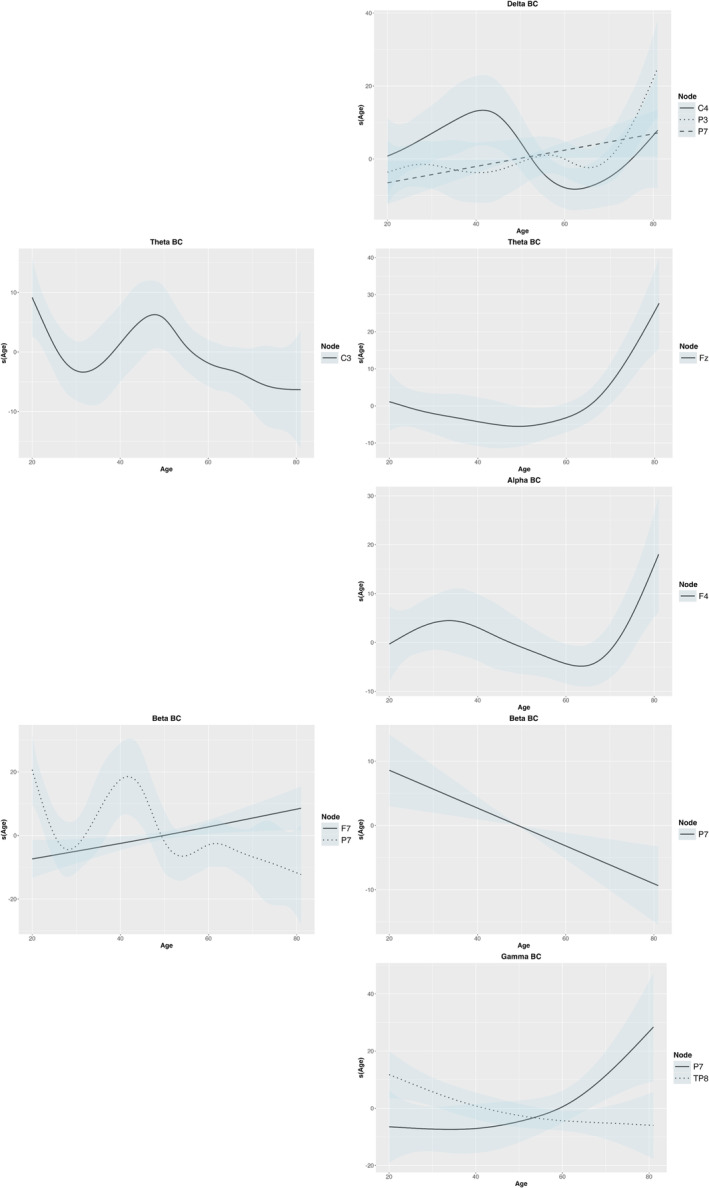
The age effect on BC values showed significant relationships across the nodes. The right side represents the shape condition, and the left side represents the binding condition.

In the delta band, three metrics showed significant age‐related patterns. The CPL metric increased linearly with age (EDF = 1.00, *F* = 4.98, *p* = 0.032). The delta hub metric revealed node‐specific variations during the binding condition (*F*_*z*_: EDF = 8.63, *F* = 2.24, *p* = 0.046; *F*_8_: EDF = 9.79, *F* = 7.84, *p* < 0.001; *FC*_4_: EDF = 10.62, *F* = 2.66, *p* = 0.020; *P*_8_: EDF = 1.85, *F* = 3.10, *p* = 0.045) (Figure 5). Regarding delta hubness in the shape condition, *O*_1_ exhibited stable trend from 20 to 60 years, followed by a sharp decline and recovery toward 80 years (EDF = 2.46, *F* = 3.84, *p* = 0.016). In contrast, *F*_7_ exhibited stable or slightly increasing hubness after 60 years, followed by a decline, indicating a shift in network centrality (EDF = 8.33, *F* = 2.77, *p* = 0.016). The bc values varied across central and parietal regions in the binding condition. *C*_4_ exhibited a slight increase post‐50 year (EDF = 4.32, *F* = 2.69, *p* = 0.038), while nodes *P*_3_ (EDF = 4.67, *F* = 2.75, *p* = 0.030) and *P*_7_ (EDF = 1.00, *F* = 4.97, *p* = 0.032) showed more complex patterns, with increase around 50 years and subsequent variability (Figure 6).

The theta bc values demonstrated complex age‐related patterns in both shape and binding conditions (Figure 6). Node *C*_3_ exhibited decline after age 20, followed by an increase toward age 50, then slight decline thereafter (EDF = 5.30, *F* = 2.53, *p* = 0.038). In the binding condition, *F*_*z*_ displayed increased bc after 60 years, highlighting its growing importance in the network with aging (EDF = 3.34, *F* = 5.24, *p* = 0.002).

In the alpha band, hubness also varied with age in the shape (*TP*_8_: EDF = 1.55, *F* = 4.63, *p* = 0.029; *P*_3_: EDF = 2.92, *F* = 3.22, *p* = 0.034) and binding (*C*_4_: EDF = 7.90, *F* = 23.19, *p* < 0.001; *O*_2_: EDF = 1.44, *F* = 4.85, *p* = 0.033) conditions (Figure 5). The alpha hubness at *P*_3_ and *TP*_8_ exhibit an increasing trend, suggesting growing importance of these areas in older ages. In contrast, *O*_2_ demonstrated an increasing trend with age. The alpha bc metric revealed a peak around 30 years at *F*_4_ (EDF = 3.91, *F* = 2.77, *p* = 0.032), followed by a decrease and a subsequent increase after 60 years, reflecting its continued relevance as aging progresses in the binding condition (Figure 6).

The beta hub values demonstrated age effects in the shape (*F*_*z*_: EDF = 2.46, *F* = 3.84, *p* = 0.016; *TP*_8_: EDF = 7.71, *F* = 10.11, *p* < 0.001; *P*_7_: EDF = 2.15, *F* = 3.43, *p* = 0.027) and binding (*FT*_7_: EDF = 1.18, *F* = 7.26, *p* = 0.010; *O*_*z*_: EDF = 3.53, *F* = 4.08, *p* = 0.007) conditions (Figure 5). Specifically, *F*_*z*_ hubness declined steeply, particularly after mid‐adulthood, while *O*_*z*_ increased with age, indicating a growing central role in the beta network in later life.

For beta bc metric, significant node‐related relationships were observed, particularly in frontal and parietal regions during the shape condition (Figure 6). Node *F*_7_ showed an increase after 50 years, reflecting enhanced centrality (EDF = 1.08, *F* = 5.53, *p* = 0.017), whereas *P*_7_ demonstrated a gradual increase starting from 40 years onwards (EDF = 6.76, *F* = 3.61, *p* = 0.004). In the binding condition, *P*_7_ exhibited more stable decline (EDF = 1.00, *F* = 9.45, *p* = 0.004).

The gamma band also presented noteworthy findings. The gamma *FC*_*z*_ (EDF = 3.53, *F* = 4.82, *p* = 0.007) and *CP*_4_ (EDF = 4.20, *F* = 16.83, *p* < 0.001) hubness remained stable until 50 years, followed by a decline, suggesting shifts in network organization, while node *F*_8_ demonstrated a variable trend (EDF = 7.36, *F* = 2.37, *p* = 0.037) in the binding condition (Figure 5). The gamma bc metric in the node *P*_7_ increased in older age (after 60 years) (EDF = 1.68, *F* = 4.10, *p* = 0.025), while *TP*_8_ showed an opposite pattern (EDF = 8.98, *F* = 3.78, *p* = 0.002) in the binding condition (Figure 6).

These results suggest that age‐related changes in network metrics are evident across multiple frequency bands, with both linear and non‐linear effects observed. The data revealed complex, and region‐specific patterns of change in brain network properties with aging. In general, there was a trend toward decreased network integrity in slow frequencies (i.e., delta and theta) as age increased, particularly after 50 years. In contrast, in higher frequency bands (i.e., beta and gamma), there was more variability, with some regions showing increased centrality.

#### 3.2.2 Segregation Metrics

Delta CC values showed a significant age effect in the shape condition, with increased values especially after age 60 (EDF = 8.58, *F* = 6.08, *p* < 0.001). Delta module values also exhibited age‐related associations in the shape condition, particularly in the left frontal areas (*F*_7_: EDF = 1.00, *F* = 5.74, *p* = 0.022; *FT*_7_: EDF = 1.00, *F* = 12.07, *p* = 0.001), with a linear decline with age (Figure 8).

**FIGURE 7:**
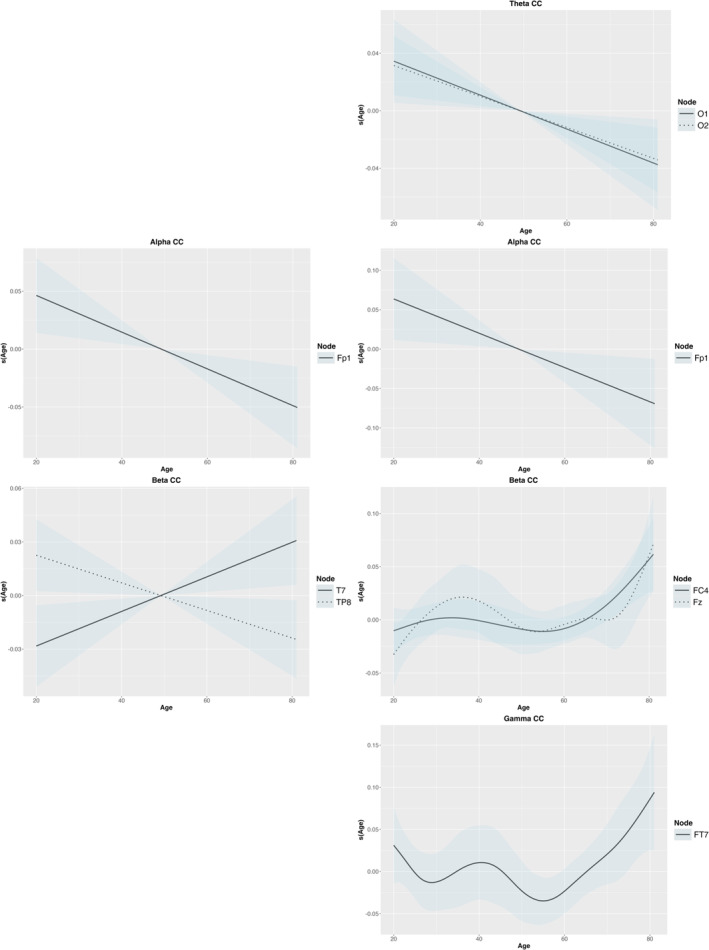
The age effect on CC values showed significant relationships across the nodes. The right side represents the shape condition, and the left side represents the binding condition.

**FIGURE 8:**
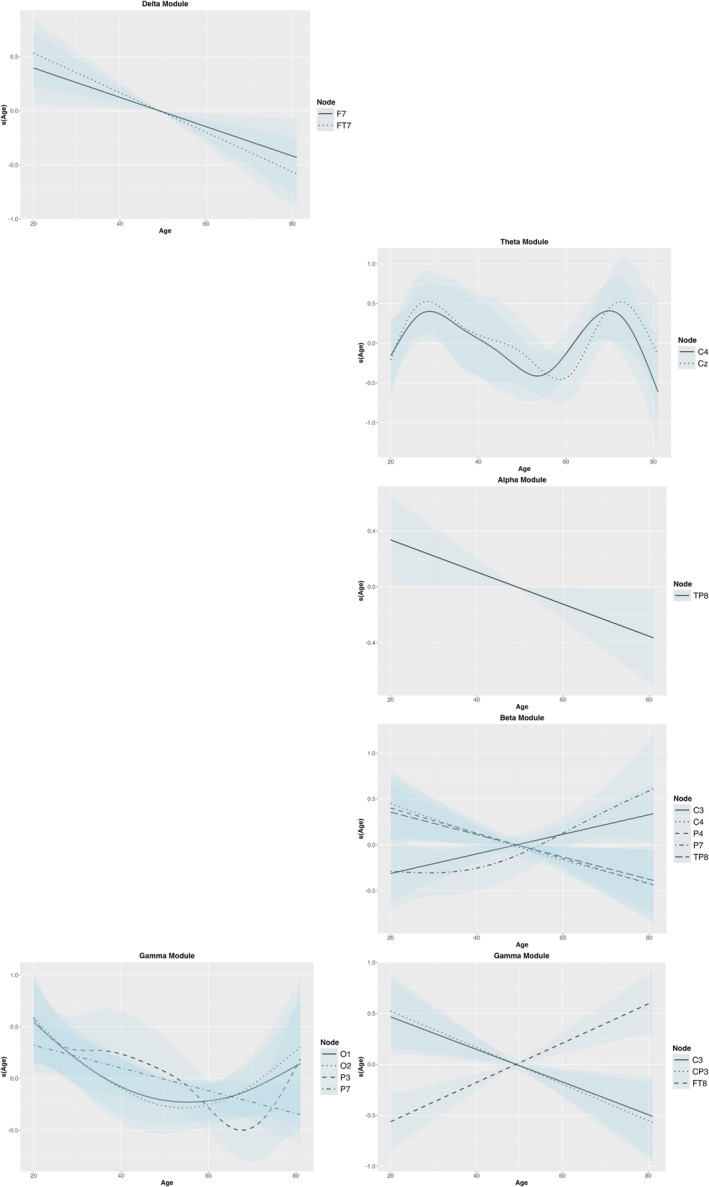
The age effect on module values showed significant relationships across the nodes. The right side represents the shape condition, and the left side represents the binding condition.

In the binding condition, theta CC displayed a significant age effect over the occipital regions in (*O*_1_: EDF = 1.00, *F* = 5.66, *p* = 0.023; *O*_2_: EDF = 1.00, *F* = 9.24, *p* = 0.004), reflecting a consistent decrease with age (Figure 7). Theta modules over central regions revealed age effects (*C*_*z*_: EDF = 6.00, *F* = 2.41, *p* = 0.042; *C*_4_: EDF = 5.31, *F* = 2.54, *p* = 0.035), with increases followed by decreases with age (Figure 8).

The alpha CC was significantly affected by age (EDF = 1.00, *F* = 5.39, *p* = 0.026), indicating a decline with age. This effect was particularly evident in the prefrontal region (*Fp1*) in both conditions (shape: EDF = 1.00, *F* = 8.17, *p* = 0.007; binding: EDF = 1.00, *F* = 5.96, *p* = 0.020) (Figure 7). Additionally, alpha modules over the node *TP*_8_ (EDF = 1.00, *F* = 4.50, *p* = 0.041) showed linear decline with age in the binding condition (Figure 8).

The beta band presented significant findings in CC and module metrics. Beta CC highlighted nodal features, especially over frontal areas (*F*_*z*_: EDF = 5.47, *F* = 2.54, *p* = 0.041; *FC*_4_: EDF = 3.20, *F* = 3.62, *p* = 0.014) in the binding condition. In the shape condition, divergent trends were observed at *T*_7_ (EDF = 1.00, *F* = 6.16, *p* = 0.018) and *TP*_8_ (EDF = 1.00, *F* = 5.03, *p* = 0.031), with *T*_7_ showing an increase and *TP*_8_ a decrease, reflecting age‐related differences in local clustering within the beta network (Figure 7). The beta module displayed linear relationships in the binding condition (*C*_3_: EDF = 1.00, *F* = 4.56, *p* = 0.039; *C*_4_: EDF = 1.15, *F* = 5.39, *p* = 0.027; *P*_4_: EDF = 1.00, *F* = 5.25, *p* = 0.028; *TP*_8_: EDF = 1.00, *F* = 5.37, *p* = 0.026; *P*_7_: EDF = 1.80, *F* = 3.35, *p* = 0.048) (Figure 8). Nodes showed mixture trends, with *C*_3_ and *P*_7_ increasing, while other nodes demonstrated decreases, reflecting varied changes in beta network modularity.

The gamma CC exhibited a significant age effect during the shape condition (*FT*_7_: EDF = 5.04, *F* = 2.47, *p* = 0.043), showing increased local clustering after age 60 (Figure 7). Gamma modules showed complex relationships during the shape (*P*_3_: EDF = 4.15, *F* = 3.55, *p* = 0.010; *P*_7_: EDF = 1.00, *F* = 5.72, *p* = 0.022; *O*_1_: EDF = 2.10, *F* = 3.21, *p* = 0.041; *O*_*z*_: EDF = 2.60, *F* = 3.12, *p* = 0.035) and binding (*FT*_8_: EDF = 1.00, *F* = 17.02, *p* < 0.001; *C*_3_: EDF = 1.05, *F* = 6.50, *p* = 0.013; *CP*_3_: EDF = 1.00, *F* = 8.66, *p* = 0.006) conditions (Figure 8). There was significant variability in modularity, decreasing in some nodes (i.e., *C*_3_, *CP*_3_ and *P*_7_), while the others exhibited more complex patterns across the age range.

The results illustrate that age has an influence on brain network metrics differently across frequency bands. Delta, theta and alpha bands showed trends toward reduction with age, particularly in specific regions, whereas beta and gamma bands reveal more complex and varied changes, with both increased and decreased, indicating nuanced age‐related transformations in brain network dynamics. These findings underscore the complex nature of age‐related changes in network segregation.

#### 3.2.3 Global Organization Metrics

The analysis revealed significant age‐related changes in SW values. Specifically, in the shape condition, alpha SW values demonstrated a linear decrease with age (EDF = 1.00, *F* = 5.06, *p* = 0.030), while beta SW values exhibited a non‐linear pattern (EDF = 2.58, *F* = 3.80, *p* = 0.017), decreasing after age 20 and increasing around age 45. Additionally, in the binding condition, gamma SW values revealed a linear decline with age (EDF = 1.00, *F* = 6.68, *p* = 0.014), highlighting a consistent reduction in gamma SW properties as individuals age.

### 3.3 Correlation Analysis Findings

Correlation analysis was conducted solely on measures that exhibited significant age effect, with the aim of reducing the number of comparisons.

#### 3.3.1 Correlations Between Integration Metrics and Cognitive Domains and Behavioural Data

In the delta band, negative correlation was found between CPL and episodic memory scores (*r* = −0.461, *p* = 0.003). Delta bc at *P*_3_ showed negative associations with MMSE scores (*r* = −0.429, *p* = 0.007) and visual memory (*r* = −0.521, *p* = 0.001). In contrast, hubness of *F*_*z*_ showed a positive correlation with MMSE scores (*r* = 0.430, *p* = 0.007).

Theta bc values at *F*_*z*_ had negative associations with visual memory (*r* = −0.480, *p* = 0.002) and visuospatial abilities (*r* = −0.632, *p* < 0.001).

Alpha hubness at *TP*_8_ was positively correlated with executive functions (*r* = 0.466, *p* = 0.003) and language skills (*r* = 0.496, *p* = 0.002). Additionally, *C*_4_ hubness showed positive associations with MMSE scores (*r* = 0.698, *p* < 0.001), visual memory (*r* = 0.616, *p* < 0.001), attention (*r* = 0.412, *p* = 0.010), executive functions (*r* = 0.439, *p* = 0.006), language skills (*r* = 0.470, *p* = 0.003) and visuospatial abilities (*r* = 0.690, *p* < 0.001). Conversely, the bc of *F*_4_ node was negatively correlated with visual memory (*r* = −0.412, *p* = 0.010) and visuospatial abilities (*r* = −0.481, *p* = 0.002).

In the beta band, *F*_*z*_ hubness showed positive associations with visual memory, language and visuospatial abilities (*r* = 0.449, *p* = 0.005; *r* = 0.421, *p* = 0.008; *r* = 0.627, *p* < 0.001, respectively). *TP*_8_ hubness revealed positive relationships with visual memory, attention, language and visuospatial abilities (*r* = 0.555, *p* < 0.001; *r* = 0.529, *p* = 0.001; *r* = 0.649, *p* < 0.001; *r* = 0.670, *p* < 0.001, respectively). Hubness at *O*_*z*_ was positively correlated with visual memory (*r* = 0.498, *p* = 0.001), attention (*r* = 0.434, *p* = 0.006) and visuospatial abilities (*r* = 0.746, *p* < 0.001).

Gamma hubness at *FC*_*z*_ and *CP*_4_ also showed positive associations with visual memory (*r* = 0.498, *p* = 0.001 and *r* = 0.517, *p* = 0.001, respectively), attention (*r* = 0.434, *p* = 0.006 and *r* = 0.628, *p* < 0.001, respectively) and visuospatial abilities (*r* = 0.746, *p* < 0.001 and *r* = 0.612, *p* < 0.001, respectively). Moreover, bc at *P*_7_ were correlated with BDI (*r* = 0.429, *p* = 0.007) and visuospatial abilities (*r* = −0.511, *p* = 0.001).

#### 3.3.2 Correlations Between Segregation Metrics and Cognitive Domains and Behavioural Data

There was a negative correlation between delta CC and the attention domain (*r* = −0.467, *p* = 0.004) and response accuracy (*r* = −0.400, *p* = 0.013). Also, beta CC at *F*_*z*_ and *FC*_4_ showed negative associations with attention domain (*r* = −0.426, *p* = 0.008 and *r* = −0.494, *p* = 0.002, respectively).

#### 3.3.3 Correlations Between Global Organizational Metrics and Cognitive Domains and Behavioural Data

The beta SW was positively associated with BDI (*r* = 0.441, *p* = 0.006), while it was negatively correlated with episodic memory (*r* = −0.426, *p* = 0.010) and attention domains (*r* = −0.459, *p* = 0.005).

## 4 Discussion

The current study aimed to delve into network characteristics in healthy aging during the working memory task and these characteristics' relation to cognitive tests. It was thought that quantifying changes in the network structure due to participation in the task of feature binding in working memory would offer comprehensive insights into healthy aging processes. Previous studies have shown mixed findings regarding the sensitivity of VSTMB tasks to aging, with some showing sensitivity (Brockmole and Logie 2013) and others suggesting insensitivity (Parra, Abrahams, Logie, and Sala 2009; Parra, Abrahams, Fabi, et al. 2009; Rhodes, Parra, and Logie 2016; Brown et al. 2017). Our findings contributed further evidence supporting complex nature of age‐related changes in brain network integration and segregation across frequency bands. In general, there was a trend toward decreased network metrics in lower frequency bands with increasing age, while higher frequency bands showed more variability. Additionally, global organization revealed frequency‐specific differences with age. These network alterations were linked to cognitive domain scores, which generally worsened with age. We will further elaborate on these findings in detail regarding network characteristics.

### 4.1 Integration Findings

More global functional integration of networks was linked to higher working memory capacity in an fMRI investigation (Stevens et al. 2012). In the delta band, we observed that as individuals age, there is an increased CPL, indicating longer paths in brain networks. This suggests reduced network efficiency, which aligns with poorer episodic memory performance. Contrary to our findings, previous studies have reported shortened CPL during visual working memory tasks (Hong et al. 2016; Hou et al. 2018; Javaid, Kumarnsit, and Chatpun 2022). However, these studies employed relatively easier tasks than ours, potentially explaining the discrepancies. Indeed, task difficulty seems to increase path length in older adults while leaving it unaffected in younger individuals (Xu et al. 2023), suggesting that cognitive load plays a role in these divergent findings. Our results also indicate that longer path lengths may reflect a disrupted network, as they correlate with reduced memory performance.

Delta, theta and alpha bc values showed similar trends in the binding condition. A node with high bc exerts significant influence on the information flow within the network. In this study, parietal delta centrality increased around the fifties, which correlated with worse global cognitive scores and visuospatial abilities. The frontal and central delta, theta and alpha centrality, on the other hand, significantly increased after age 60, which were also linked to poorer visual memory and visuospatial abilities. These results suggest that aging impacts the network's information flow, with increased values in older individuals potentially reflecting compensation, as suggested by the posterior–anterior shift in aging (PASA) theory, which posits a compensatory shift from posterior to anterior regions in older adults (Festini, Zahodne, and Reuter‐Lorenz 2018). This theory also confirmed by other integration metric, hubness, revealing recovered frontal delta hubness and declined parietal/occipital delta hubness in older age. The frontal hubness was also related to global cognitive scores. Furthermore, considering the delta CPL and bc results together, we can suggest that the ineffective network reorganization in older individuals might lead to increased path lengths (Xiang, Wang, and You 2023).

In the shape condition, the theta bc displayed complex age‐related effects in the central area, exhibiting increased centrality until middle age, followed by a decrease, and slight recovery around the sixties. This aligns with the compensation‐related utilization of neural circuits hypothesis (CRUNCH) model, suggesting that older adults exhibit compensatory age‐related overactivation. This model posits that at relatively low levels of task demands, older adults are more likely to show increased activation compared to younger adults (e.g., in the shape condition of the current study) (Festini, Zahodne, and Reuter‐Lorenz 2018). This also supports the integrative role of theta frequency in working memory tasks, attributed to compensatory activation, which is characteristic of normal aging (Phillips and Andrés 2010).

In the alpha band, we observed that hubness increased in the temporo‐parietal regions with age, particularly during the shape condition, and was associated with better executive function and language skills. During the binding condition, hubness exhibited a stable trend in the right central area until the sixties, followed by a sharp decline. Contrary, hubness of the right occipital region increased with age. These shifts were associated with cognitive domains such as visual memory, attention and executive function, suggesting that age‐related network reorganization affects multiple cognitive functions. Studies have underscored the importance of hubs in promoting effective neural communication and information integration, especially for successful cognitive performance (Bassett and Bullmore 2009; van den Heuvel et al. 2010; Cole et al. 2013; van den Heuvel and Sporns 2013). Moreover, strong alpha activity has been associated with working memory, information integration and cortical inhibition (Klimesch 2012). This activity serves as an indicator of an inhibition mechanism that prioritizes information processing (Hinault et al. 2021). According to the scaffolding theory of aging and cognition (STAC), older adults with less neural degeneration and a greater ability to use compensatory scaffolds will exhibit better cognitive performance than those who cannot recruit these scaffolds (Festini, Zahodne, and Reuter‐Lorenz 2018; Oosterhuis et al. 2023). Our results support this theory, suggesting that posterior regions may serve as scaffolds to maintain cognitive function in aging, especially because our correlation results indicated better performances. However, the decline in central alpha activity may reflect the challenges older adults face in maintaining task performance as working memory load increases (Chen and Huang 2016).

In the beta band, during the shape condition, hubness of midline frontal, parietal and temporo‐parietal regions decreased after age 60, correlating with performance in visual memory, attention, language skills and visuospatial abilities. Beta activity plays a crucial role in filtering irrelevant information and maintaining high working memory performance (Zanto and Gazzaley 2009). Decreases in beta hubness may reflect less effective inhibition processes in older adults, leading to altered network organization. In contrast, during the binding condition, beta hubness of the left frontotemporal area increased, while occipital hubness decreased, correlating with visual memory, attention and visuospatial abilities. Earlier studies have reported that connector hubs adjust the connectivity of their neighbours to be more modular, while facilitating task‐appropriate information integration between communities (Bertolero et al. 2018). This phenomenon improves global modularity and cognitive performance. A study utilizing the *N*‐back task with a mixed group of young and middle‐aged participants discovered that connector hubs increased with working memory load (Stanley et al. 2014). Working memory networks are characterized by high‐frequency oscillations in the right frontotemporal areas and in parieto‐occipital connections, which may underlie visual information processing during the presentation and retrieval of visual stimuli (Toppi et al. 2018). Increased frontotemporal hubness may be related to working memory load and information encoding, which has been shown to enhance temporal beta activity (Chen and Huang 2016; Toppi et al. 2018). These changes are consistent with studies showing that beta frequency networks become more dynamic under increased task difficulty (Chen and Huang 2016; Toppi et al. 2018).

Beta centrality also showed age‐related changes, with increases in left lateral frontal areas and decreases in the parietal area after age 40. These regional differences have been reported previously, showing that age‐related declines in perceptual processing in the posterior cortex may result in increased compensatory activation in higher order anterior processing centres (Wijeakumar, Magnotta, and Spencer 2017). According to Wijeakumar, Magnotta, and Spencer's (2017) findings, the frontal cortex attempts to augment working memory representations in the anterior cortex to compensate for the age‐related declines in the posterior regions. This pattern aligns with the hemispheric asymmetry reduction in older adults (HAROLD) model, which suggests that elderly individuals exhibit bilateral frontal activity, whereas younger adults show unilateral (e.g., right‐lateralized) activation of frontal regions during working memory tasks (Festini, Zahodne, and Reuter‐Lorenz 2018).

In feature binding tasks and working memory load, gamma activity is crucial for reflecting performance (Tseng et al. 2016). Indeed, gamma hubs primarily showed age effects during the binding condition, with the frontocentral and right centro‐parietal areas exhibiting stable values until the sixties, followed by a sharp decline. These changes were linked to visual memory, attention and visuospatial abilities. The gamma hubness of right frontal region exhibited a more complex relationship with age, decreasing through the forties and then normalizing. Gamma activity over the frontal, frontotemporal and central areas has been associated with information storage and retrieval (Toppi et al. 2018). Taken together, both gamma and beta hubness suggested that inhibition and memory processes can be compromised with age.

Lastly, younger participants showed higher gamma bc in the right temporo‐parietal, while left lateral parietal bcs were more pronounce in older ages, especially after the sixties. Earlier studies have consistently showed right hemispheric dominance, especially over the parietal cortex, during binding tasks, correlating with visual working memory capacity and accuracy (Ashbridge, Cowey, and Wade 1999; Shafritz, Gore, and Marois 2002; Vogel and Machizawa 2004; Todd and Marois 2004; Sauseng et al. 2009; Honkanen et al. 2015). Our correlational findings further confirmed this right hemispheric dominance, by showing relations of higher left lateral parietal gamma bc with poorer visuospatial abilities and higher depressive symptoms.

In summary, our study revealed distinct age‐related changes in brain network integration across different frequency bands. Older adults displayed increased path lengths, reflecting reduced network efficiency and poorer memory performance, a shift from posterior to anterior brain regions with age, consistent with the PASA theory. Thus, middle‐aged individuals maintained more efficient network integration, while older adults experienced declines in network efficiency and cognitive performance.

### 4.2 Segregation Findings

Our analysis of CC in the delta band revealed that elderly participants, particularly those over 60, exhibited higher values, which were related with lower response accuracy and attention scores. These findings are consistent with previous studies linking delta CC and attention deficits (Chua et al. 2017; Li et al. 2019). In contrast, theta CCs over the occipital regions gradually decreased with age, reflecting changes in local network integration. CC measures local clustering (i.e., global segregation), which is important for maintaining information processing within a network (Wodeyar and Srinivasan 2018). Increased theta CC has been shown in young participants during target processing in the *oddball* task (Bola and Sabel 2015). Additionally, numerous studies have demonstrated an increase in theta network segregation with high working memory load (Sauseng et al. 2005; Langer, Pedroni, and Jäncke 2013; Dai et al. 2017).

Module analysis also showed age‐related changes, with delta modularity decreasing over the left lateral frontal and frontotemporal regions. Meanwhile, central theta modularity showed a more dynamic pattern, increasing until the thirties, declining through the fifties, rose again and then declined after the seventies. It is well‐established that slow‐frequency networks play an important role in top‐down cognitive processing (Fiebelkorn, Saalmann, and Kastner 2013; Dugué, Marque, and VanRullen 2015; Tobe et al. 2023). Studies have shown that slow frequencies favour flexible encoding of task‐specific events in humans (Johnson et al. 2019). In particular, frontal delta activity is associated with internal priority switch (de Vries et al. 2018). On the other hand, the theta band plays a crucial role in working memory, as it is associated with object retention and the retrieval of visual information (Mizuhara and Yamaguchi 2007; Sauseng et al. 2007). The topology of theta band network undergoes prompt and temporary reorganization, which is related to perceptual and cognitive processing (Bola and Sabel 2015). Our findings suggest that older adults experience greater network disruption in the delta band, while middle‐aged adults may use central regions as compensatory scaffolds in line with the STAC model.

In the alpha band, we observed significant decreases in CC over the prefrontal regions and reductions in modularity over the right temporo‐parietal regions with age. The brain integrates specialized modules into a functional network to maintain memories within the prefrontal cortex, directing attention toward representations in the brain, and within the sensory, parietal and temporal areas (LaRocque et al. 2013; Ester, Sprague, and Serences 2015; Wolff et al. 2015; Bettencourt and Xu 2016; Wodeyar and Srinivasan 2018). Alpha activity supports working memory, information integration and cortical inhibition (Klimesch 2012), with decreased segregation suggesting that older adults have less specialized networks, leading to cognitive inefficiencies. Hinault et al. (2021) also found that lower structural clustering in older adults correlated with reduced clustering and efficiency of task‐related functional network in the alpha band. Thus, it can be assumed that information integration, attention and inhibition‐related networks becomes less segregated, more integrated with aging.

Beta CC values increased with age in the left temporal area but decreased in the right temporo‐parietal regions during the shape condition. This pattern is consistent with findings showing that younger adults exhibit higher beta CC during demanding cognitive tasks, particularly in posterior regions, while older adults show no such increases (Hou et al. 2018). In the binding condition, midline frontal and right frontocentral CC values increased in younger ages, decreased during middle ages and then increased again after the sixties. These values were also found to be related to attention scores. Working memory models attribute the control function to the frontal regions (D'Esposito and Postle 2015). Similarly, a recent study found enhanced beta powers at the right‐inferior frontal gyrus specifically in response to trials with strong response conflict and switching (Daniel et al. 2023). These results suggest that cognitive control may change dynamically with aging, leading to response uncertainty.

Beta modules over right central, temporo‐parietal and parietal regions decreased with age, whereas left central and parietal increased during the binding condition. It is worth noting that beta activity serves as an essential indicator of interneuronal communication in functional working memory networks (Guevara et al. 2018), and a key characteristic of beta networks is that their modularity tends to decrease with increased mental workload (Zhang et al. 2017; Kakkos et al. 2019). This decrease in modularity is typically associated with the brain adopting a more efficient but less clustered network topology to manage increased cognitive demands (Kitzbichler et al. 2011). Beta frequency has also been associated with attentional deficiencies in elderly individuals (Teng et al. 2018). As beta frequency is involved in filtering irrelevant information and maintaining focused attention, the reduced segregation of beta networks in older adults may contribute to deficits in attention and working memory. Our findings highlight that as individuals age, beta oscillations play an increasingly important role in the dynamic reconfiguration of brain networks to support cognitive function (Kitzbichler et al. 2011; Xu et al. 2023).

Gamma frequency, which is initially related to segregation in simpler tasks, transitions to become associated with integration as task difficulty increases. Indeed, we observed an age effect on integration metrics only during the binding condition, while segregation metrics were affected by age in both conditions. During the shape condition, gamma modules were less prominent over the left parietal and occipital locations in the middle age. This aligns with recent findings that occipital‐parietal gamma oscillations enhance memory performance, highlighting their role in critical working memory processes (Wischnewski et al. 2024). It has been also suggested that the parieto‐occipital connection observed in high‐frequency oscillations may underlie visual information processing during the presentation and retrieval of visual stimuli (Toppi et al. 2018). In the binding condition, older individuals exhibited fewer modules in the left central and centro‐parietal regions but showed an increase in right frontotemporal modules. Additionally, gamma CC over the left frontotemporal areas rose during the forties, followed by a decline and then a sharp increase after the fifties. This pattern can be understood in light of the STAC model, which proposes that aging involves compensatory overactivation in regions suited for higher level processes, alongside underactivation in more specialized areas (Festini, Zahodne, and Reuter‐Lorenz 2018). Occipital gamma synchronization has also been associated with participants' performance during the memory stages (Osipova et al. 2006). Overall, these findings indicate that with age, differentiation in the modularity of the brain network system at the level of frequencies becomes evident.

In summary, in line with the dedifferentiation hypothesis in aging—which implies greater similarity or reduced distinctiveness of neural responses (Festini, Zahodne, and Reuter‐Lorenz 2018)—our findings indicated a reduction in brain network segregation in the elderly individuals. Previous research has established that global network segregation is directly linked to aging and cognitive decline (Rakesh, Fernando, and Mansour 2020; Pedersen et al. 2021). Our current study provides further evidence of this decreased segregation, highlighting frequency‐based differences and age effect. We conclude that segregation in delta, theta and gamma frequency bands better reflects age‐related differences, with these segregation changes becoming most evident after middle age.

### 4.3 Global Organization Findings

Cognitive functions are often characterized by increased SW organization of functional networks, which optimizes cognitive information processing (Stam and van Dijk 2002; Pijnenburg et al. 2004). Studies have shown that SW features of brain network change topologically under different cognitive loads (Braun et al. 2015; Cao et al. 2016; Liang, Pertzov, et al. 2016; Dai et al. 2017). During visual working memory tasks, alpha and beta networks tend to exhibit more SW structure compared to other frequency bands (Palva et al. 2010). Consistent with these findings, our study revealed group differences in SW of these frequency bands. Specifically, alpha SW characteristics declined with age, whereas the beta SW showed a more complex pattern, with a decline after age 20 followed by an increase at the age 40. The reduction in alpha SW in elderly is expected, as previously shown *N*‐back task (Hou et al. 2018). However, the observed pattern in beta band is novel, as earlier studies did not report this nuanced relationship (Hong et al. 2016; Hou et al. 2018).

Previously, beta SW has been shown to increase in elderly during cognitive tasks compared to resting state, whereas a similar increase is absent in the younger group (Hou et al. 2018). Moreover, higher beta connectivity has been linked to lower cognitive reserve (López et al. 2014), which may explain the increase in beta SW observed in older individuals. In line with this, we further showed that higher beta SW was associated with worse episodic memory and attention scores, as well as increased depressive symptoms. Given that beta band has been reported as more sensitive to aging than other bands (Gaál et al. 2010; Knyazev, Volf, and Belousova 2015; Hong et al. 2016; Wang et al. 2017; Arif et al. 2020), it is understandable to see this complex age effect on beta SW. In contrast, gamma SW decreased with age in the binding condition, reflecting its role in high‐level feature binding processes and its association with performance in this condition. The decline in gamma SW with age is consistent with the notion that younger individuals typically exhibit more optimal gamma network organization, leading to better task performance.

### 4.4 Neuropsychological Profiles

Healthy aging typically involves a decline in cognitive performance, particularly in episodic memory and executive functioning (Buckner 2004). Previous studies have consistently reported that working memory and attention functions are among the most vulnerable areas to the effects of aging (Cohen, Marsiske, and Smith 2019). Consistent with these findings, the current study found differences in cognitive domains as a function of age. The pronounced effect of age on episodic memory and attention supports the notion that these domains are vulnerable to aging, with performance declines becoming evident after the age of 50. Visual memory also exhibited non‐linear effect, with a marked decline after age 60, which may reflect age‐related changes in the neural circuits underlying visual information processing.

Many cross‐sectional studies have shown that crystallized abilities tend to peak around the age of 60 and then gradually decline until age 80. In contrast, fluid abilities, which involve cognitive processing and manipulation of information, decline continuously from around age 20 to 80 (Salthouse 2010a, 2010b; Lezak et al. 2012; Murman 2015). Indeed, language skills demonstrated a similar pattern, increasing until around age 45 before beginning to decline. Likewise, visuospatial abilities showed a sharp decline after age 70. Additionally, executive functions remained stable until middle age, they began to decline thereafter, aligning with previous studies reporting executive functions tend to decline, especially after the age of 70 (Salthouse 2010a, 2010b; Lezak et al. 2012; Hamasaki et al. 2018).

## 5 Conclusion

In this study, we demonstrated that changes in cognitive network properties, rather than neuropsychological assessments alone, serve as sensitive markers of age‐related changes. More importantly, we gained valuable insights into modifications in brain networks with age, by examining individuals, who were deemed healthy based on cognitive tests' norm values, across a wide age range using EEG during a working memory task. Our graph analysis, assessed within the framework of integration, segregation and global organization, indicated that middle‐aged individuals experience more pronounced and efficient changes in network architecture. However, this effectiveness seems to vanish or show compensatory mechanisms in elderly individuals.

Earlier studies have shown that network structure can become more integrated or segregated depending on task demands, although this trade‐off is not general for all tasks (Cohen and D'Esposito 2016). Our results align with previous research suggesting that network structure is influenced by task difficulty (Kitzbichler et al. 2011; Cohen et al. 2014; Ren et al. 2017; Liang, Zou, He, and Yang 2016), with increased integration observed in more challenging tasks, such as the colour‐shape binding, and greater segregation in less demanding tasks. These changes in network architecture are associated with performance outcomes, supporting the notion that network efficiency and integration are crucial for cognitive function (Cohen et al. 2014; Liang, Zou, He, and Yang 2016).

This study is the first to show the effects of age on working memory network architecture. However, it has several limitations to be addressed. Even though it encompassed a broad age range without focusing solely on extreme ages, it is important to note that age‐related effects in cross‐sectional data represent only proxies of long‐term changes and cannot establish causal relationships regarding brain network changes over time. Future longitudinal studies assessing the differentiation of working memory networks with age are necessary to directly examine the impacts of maturational changes. Additionally, the current study's results should be interpreted with caution due to the small sample size. Although the sample size was adequate for statistical analysis, future studies should aim to recruit more participants to increase statistical power.

Understanding the changing brain network systems in healthy aging is crucial for comprehending and preventing pathological aging. There are some interventions available or being developed that can improve cognitive function, such as cognitive training, neuromodulation and pharmacological approaches. However, preventing, slowing and reversing the negative effects of cognitive aging remains a challenge. Therefore, further studies are needed to elucidate healthy aging‐related changes and develop effective interventions.

## Author Contributions

**Ezgi Fide:** conceptualization, data curation, formal analysis, methodology, visualization, writing – original draft. **Emre Bora:** conceptualization, methodology, project administration, supervision, writing – review and editing. **Görsev Yener:** conceptualization, project administration, resources, supervision, writing – review and editing.

## Conflicts of Interest

The authors declare no conflicts of interest.

### Peer Review

The peer review history for this article is available at https://www.webofscience.com/api/gateway/wos/peer‐review/10.1111/ejn.70001.

## Data Availability

The data that support the findings of this study are openly available in figshare repository at https://doi.org/10.6084/m9.figshare.25883665.v1.

## References

[ejn70001-bib-0001] Achard, S. , R. Salvador , B. Whitcher , J. Suckling , and E. Bullmore . 2006. “A Resilient, Low‐Frequency, Small‐World Human Brain Functional Network With Highly Connected Association Cortical Hubs.” Journal of Neuroscience 26, no. 1: 63–72. 10.1523/JNEUROSCI.3874-05.2006. 16399673 PMC6674299

[ejn70001-bib-0002] Alavash, M. , C. M. Thiel , and C. Gießing . 2016. “Dynamic Coupling of Complex Brain Networks and Dual‐Task Behavior.” NeuroImage 129: 233–246. 10.1016/j.neuroimage.2016.01.028. 26803061

[ejn70001-bib-0003] Arif, Y. , R. K. Spooner , A. I. Wiesman , C. M. Embury , A. L. Proskovec , and T. W. Wilson . 2020. “Modulation of Attention Networks Serving Reorientation in Healthy Aging.” Aging (Albany NY) 12, no. 13: 12582–12597. 10.18632/aging.103515. 32584264 PMC7377885

[ejn70001-bib-0004] Arul, S. M. , G. Senthil , S. Jayasudha , A. Alkhayyat , K. Azam , and R. Elangovan . 2023. “Graph Theory and Algorithms for Network Analysis.” E3S Web of Conferences 399: 08002. 10.1051/e3sconf/202339908002.

[ejn70001-bib-0005] Ashbridge, E. , A. Cowey , and D. Wade . 1999. “Does Parietal Cortex Contribute to Feature Binding?” Neuropsychologia 37: 999–1004. 10468364 10.1016/s0028-3932(98)00160-2

[ejn70001-bib-0006] Baddeley, A. 1986. Working Memory. Oxford: Clarendon.

[ejn70001-bib-0007] Basak, C. , and P. Verhaeghen . 2011. “Aging and Switching the Focus of Attention in Working Memory: Age Differences in Item Availability but Not in Item Accessibility.” Journals of Gerontology. Series B, Psychological Sciences and Social Sciences 66, no. 5: 519–526. 10.1093/geronb/gbr028. 21571704 PMC3155026

[ejn70001-bib-1001] Bassett, D. S. , and E. T. Bullmore . 2009. “Human Brain Networks in Health and Disease.” Current Opinion in Neurology 22, no. 4: 340–347. 10.1097/WCO.0b013e32832d93dd. 19494774 PMC2902726

[ejn70001-bib-0008] Bassett, D. S. , and E. T. Bullmore . 2017. “Small‐World Brain Networks Revisited.” Neuroscientist 23: 499–516. 27655008 10.1177/1073858416667720 PMC5603984

[ejn70001-bib-0009] Beck, A. T. , C. H. Ward , M. Mendelson , J. Mock , and J. Erbaugh . 1961. “An Inventory for Measuring Depression.” Archives of General Psychiatry 4, no. 6: 561–571. 13688369 10.1001/archpsyc.1961.01710120031004

[ejn70001-bib-0010] Bell, A. J. , and T. J. Sejnowski . 1997. “The “Independent Components” of Natural Scenes Are Edge Filters.” Vision Research 37, no. 23: 3327–3338. 10.1016/s0042-6989(97)00121-1. 9425547 PMC2882863

[ejn70001-bib-0011] Benton, A. L. , N. R. Varney , and K. D. Hamsher . 1978. “Visuospatial Judgment. A Clinical Test.” Archives of Neurology 35: 364–367. 655909 10.1001/archneur.1978.00500300038006

[ejn70001-bib-0012] Bertolero, M. A. , B. T. T. Yeo , D. S. Bassett , and M. D'Esposito . 2018. “A Mechanistic Model of Connector Hubs, Modularity and Cognition.” Nature Human Behaviour 2, no. 10: 765–777. 10.1038/s41562-018-0420-6. PMC6322416 30631825

[ejn70001-bib-0013] Bettencourt, K. C. , and Y. Xu . 2016. “Decoding the Content of Visual Short‐Term Memory Under Distraction in Occipital and Parietal Areas.” Nature Neuroscience 19, no. 1: 150–157. 10.1038/nn.4174. 26595654 PMC4696876

[ejn70001-bib-0014] Betzel, R. F. , and D. S. Bassett . 2017. “Multi‐Scale Brain Networks.” NeuroImage 160: 73–83. 10.1016/j.neuroimage.2016.11.006. 27845257 PMC5695236

[ejn70001-bib-1002] Bola, M. , and B. A. Sabel . 2015. “Dynamic Reorganization of Brain Functional Networks During Cognition.” NeuroImage 114: 398–413. 10.1016/j.neuroimage.2015.03.057. 25828884

[ejn70001-bib-0015] Bopp, K. L. , and P. Verhaeghen . 2020. “Aging and *n*‐Back Performance: A Meta‐Analysis.” Journals of Gerontology. Series B, Psychological Sciences and Social Sciences 75, no. 2: 229–240. 10.1093/geronb/gby024. 31943115

[ejn70001-bib-0016] Borella, E. , B. Carretti , C. Cornoldi , and R. De Beni . 2007. “Working Memory, Control of Interference and Everyday Experience of Thought Interference: When Age Makes the Difference.” Aging Clinical and Experimental Research 19, no. 3: 200–206. 10.1007/BF03324690. 17607087

[ejn70001-bib-0017] Braun, U. , A. Schäfer , H. Walter , et al. 2015. “Dynamic Reconfiguration of Frontal Brain Networks During Executive Cognition in Humans.” Proceedings of the National Academy of Sciences of the United States of America 112, no. 37: 11678–11683. 10.1073/pnas.1422487112. 26324898 PMC4577153

[ejn70001-bib-0018] Brockmole, J. R. , and R. H. Logie . 2013. “Age‐Related Change in Visual Working Memory: A Study of 55,753 Participants Aged 8‐75.” Frontiers in Psychology 4: 12. 10.3389/fpsyg.2013.00012. 23372556 PMC3557412

[ejn70001-bib-0019] Brockmole, J. R. , M. A. Parra , S. Della Sala , and R. H. Logie . 2008. “Do Binding Deficits Account for Age‐Related Decline in Visual Working Memory?” Psychonomic Bulletin & Review 15, no. 3: 543–547. 10.3758/pbr.15.3.543. 18567252

[ejn70001-bib-0020] Brown, L. A. , and J. R. Brockmole . 2010. “The Role of Attention in Binding Visual Features in Working Memory: Evidence From Cognitive Ageing.” Quarterly Journal of Experimental Psychology 63, no. 10: 2067–2079. 10.1080/17470211003721675. 20446186

[ejn70001-bib-0021] Brown, L. A. , E. H. Niven , R. H. Logie , S. Rhodes , and R. J. Allen . 2017. “Visual Feature Binding in Younger and Older Adults: Encoding and Suffix Interference Effects.” Memory 25, no. 2: 261–275. 10.1080/09658211.2016.1156705. 26983098

[ejn70001-bib-0022] Buckner, R. L. 2004. “Memory and Executive Function in Aging and AD: Multiple Factors That Cause Decline and Reserve Factors That Compensate.” Neuron 44: 195–208. 15450170 10.1016/j.neuron.2004.09.006

[ejn70001-bib-0023] Cangöz, B. , E. Karakoç , and K. Selekler . 2006. “Saat Çizme Testinin 50 yaş ve Üzeri Türk Yetişkin ve Yaşlı Örneklemi Üzerindeki Norm Belirleme ve Geçerlik‐Güvenirlik Çalışmaları.” Turkish Journal of Geriatrics 9, no. 3: 136–142.

[ejn70001-bib-0024] Cangöz, B. , E. Karakoc , and K. Selekler . 2009. “Trail Making Test: Normative Data for Turkish Elderly Population by Age, Sex and Education.” Journal of the Neurological Sciences 283, no. 1–2: 73–78. 19264326 10.1016/j.jns.2009.02.313

[ejn70001-bib-0025] Cao, M. , H. Huang , Y. Peng , Q. Dong , and Y. He . 2016. “Toward Developmental Connectomics of the Human Brain.” Frontiers in Neuroanatomy 10: 25. 10.3389/fnana.2016.00025. 27064378 PMC4814555

[ejn70001-bib-0026] Cecchini, M. A. , M. S. Yassuda , P. Squarzoni , et al. 2021. “Deficits in Short‐Term Memory Binding Are Detectable in Individuals With Brain Amyloid Deposition in the Absence of Overt Neurodegeneration in the Alzheimer's Disease Continuum.” Brain and Cognition 152: 105749. 10.1016/j.bandc.2021.105749. 34022637

[ejn70001-bib-0027] Chen, M. , and M. W. Deem . 2015. “Development of Modularity in the Neural Activity of Children's Brains.” Physical Biology 12, no. 1: 016009. 10.1088/1478-3975/12/1/016009. 25619207 PMC4489707

[ejn70001-bib-0028] Chen, Y. , and X. Huang . 2016. “Modulation of Alpha and Beta Oscillations During an n‐Back Task With Varying Temporal Memory Load.” Frontiers in Psychology 6: 2031. 10.3389/fpsyg.2015.02031. 26779113 PMC4705233

[ejn70001-bib-0029] Chua, B. L. , Z. Dai , N. Thakor , A. Bezerianos , and Y. Sun . 2017. “Connectome Pattern Alterations With Increment of Mental Fatigue in One‐Hour Driving Simulation.” In 2017 39th Annual International Conference of the IEEE Engineering in Medicine and Biology Society (EMBC), 4355–4358. Jeju, South Korea: IEEE. 10.1109/EMBC.2017.8037820. 29060861

[ejn70001-bib-1003] Cole, M. W. , J. R. Reynolds , J. D. Power , G. Repovs , A. Anticevic , and T. S. Braver . 2013. “Multi‐task Connectivity Reveals Flexible Hubs for Adaptive Task Control.” Nature Neuroscience 16, no. 9: 1348–1355. 10.1038/nn.3470. 23892552 PMC3758404

[ejn70001-bib-0030] Cohen, J. R. , and M. D'Esposito . 2016. “The Segregation and Integration of Distinct Brain Networks and Their Relationship to Cognition.” Journal of Neuroscience 36, no. 48: 12083–12094. 10.1523/JNEUROSCI.2965-15.2016. 27903719 PMC5148214

[ejn70001-bib-0031] Cohen, J. R. , C. L. Gallen , E. G. Jacobs , T. G. Lee , and M. D'Esposito . 2014. “Quantifying the Reconfiguration of Intrinsic Networks During Working Memory.” PLoS ONE 9: e106636. 25191704 10.1371/journal.pone.0106636 PMC4156328

[ejn70001-bib-0032] Cohen, R. A. , M. M. Marsiske , and G. E. Smith . 2019. “Neuropsychology of Aging.” Handbook of Clinical Neurology 167: 149–180. 10.1016/B978-0-12-804766-8.00010-8. 31753131

[ejn70001-bib-0033] Dai, Z. , J. de Souza , J. Lim , et al. 2017. “EEG Cortical Connectivity Analysis of Working Memory Reveals Topological Reorganization in Theta and Alpha Bands.” Frontiers in Human Neuroscience 11: 237. 10.3389/fnhum.2017.00237. 28553215 PMC5427143

[ejn70001-bib-0034] Dancey, C. P. , and J. Reidy . 2007. Statistics Without Maths for Psychology. London, UK: Pearson/Prentice Hall.

[ejn70001-bib-0035] Daniel, P. L. , J. J. Bonaiuto , S. Bestmann , A. R. Aron , and S. Little . 2023. “High Precision Magnetoencephalography Reveals Increased Right‐Inferior Frontal Gyrus Beta Power During Response Conflict.” Cortex 158: 127–136. 10.1016/j.cortex.2022.10.007. 36521374 PMC9840697

[ejn70001-bib-0036] de Boor, C. 1978. A Practical Guide to Splines. New York, NY: Springer‐Verlag New York.

[ejn70001-bib-0037] de Vries, I. E. J. , J. van Driel , M. Karacaoglu , and C. N. L. Olivers . 2018. “Priority Switches in Visual Working Memory Are Supported by Frontal Delta and Posterior Alpha Interactions.” Cerebral Cortex 28, no. 11: 4090–4104. 10.1093/cercor/bhy223. 30215669 PMC6188546

[ejn70001-bib-0038] Della Sala, S. , I. Kozlova , A. Stamate , and M. A. Parra . 2018. “A Transcultural Cognitive Marker of Alzheimer's Disease.” International Journal of Geriatric Psychiatry 33, no. 6: 849–856. 10.1002/gps.4610. 27805729

[ejn70001-bib-0039] D'Esposito, M. , and B. R. Postle . 2015. “The Cognitive Neuroscience of Working Memory.” Annual Review of Psychology 66: 115–142. 10.1146/annurev-psych-010814-015031. PMC4374359 25251486

[ejn70001-bib-0040] Dominici, F. , A. McDermott , S. L. Zeger , and J. M. Samet . 2002. “On the Use of Generalized Additive Models in Time‐Series Studies of Air Pollution and Health.” American Journal of Epidemiology 156, no. 3: 193–203. 10.1093/aje/kwf062. 12142253

[ejn70001-bib-0041] Dugué, L. , P. Marque , and R. VanRullen . 2015. “Theta Oscillations Modulate Attentional Search Performance Periodically.” Journal of Cognitive Neuroscience 27: 945–958. 10.1162/jocn_a_00755. 25390199

[ejn70001-bib-0042] Efron, B. 2007. “Size, Power and False Discovery Rates.” Annals of Statistics 35, no. 4: 1351–1377.

[ejn70001-bib-0043] Emek Savaş, D. D. , D. Yerlikaya , G. Yener , and Ö. Öktem Tanör . 2020. “Validity, Reliability and Normative Data of the Stroop Test Çapa Version.” Türk Psikiyatri Dergisi 31, no. 1: 9–21. 10.5080/u23549. 32594475

[ejn70001-bib-0044] Eriksson, J. , E. K. Vogel , A. Lansner , F. Bergström , and L. Nyberg . 2015. “Neurocognitive Architecture of Working Memory.” Neuron 88, no. 1: 33–46. 10.1016/j.neuron.2015.09.020. 26447571 PMC4605545

[ejn70001-bib-0045] Ester, E. F. , T. C. Sprague , and J. T. Serences . 2015. “Parietal and Frontal Cortex Encode Stimulus‐Specific Mnemonic Representations During Visual Working Memory.” Neuron 87, no. 4: 893–905. 10.1016/j.neuron.2015.07.013. 26257053 PMC4545683

[ejn70001-bib-0046] Farahani, F. V. , W. Karwowski , and N. R. Lighthall . 2019. “Application of Graph Theory for Identifying Connectivity Patterns in Human Brain Networks: A Systematic Review.” Frontiers in Neuroscience 13: 585. 10.3389/fnins.2019.00585. 31249501 PMC6582769

[ejn70001-bib-0047] Festini, S. B. , L. Zahodne , and P. A. Reuter‐Lorenz . 2018. “Theoretical Perspectives on Age Differences in Brain Activation: HAROLD, PASA, CRUNCH—How Do They STAC Up?” In Oxford Research Encyclopedia of Psychology, edited by B. G. Knight . Oxford: Oxford University Press. 10.1093/acrefore/9780190236557.013.400.

[ejn70001-bib-0048] Fiebelkorn, I. C. , Y. B. Saalmann , and S. Kastner . 2013. “Rhythmic Sampling Within and Between Objects Despite Sustained Attention at a Cued Location.” Current Biology 23: 2553–2558. 10.1016/j.cub.2013.10.063. 24316204 PMC3870032

[ejn70001-bib-0049] Fjell, A. M. , and K. B. Walhovd . 2010. “Structural Brain Changes in Aging: Courses, Causes and Cognitive Consequences.” Reviews in the Neurosciences 21: 187–221. 10.1515/REVNEURO.2010.21.3.187. 20879692

[ejn70001-bib-0050] Gaál, Z. A. , R. Boha , C. J. Stam , and M. Molnár . 2010. “Age‐Dependent Features of EEG‐Reactivity—Spectral, Complexity, and Network Characteristics.” Neuroscience Letters 479, no. 1: 79–84. 10.1016/j.neulet.2010.05.037. 20560166

[ejn70001-bib-0051] Geerligs, L. , N. M. Maurits , R. J. Renken , and M. M. Lorist . 2014. “Reduced Specificity of Functional Connectivity in the Aging Brain During Task Performance.” Human Brain Mapping 35, no. 1: 319–330. 10.1002/hbm.22175. 22915491 PMC6869200

[ejn70001-bib-0052] Geerligs, L. , E. Saliasi , N. M. Maurits , R. J. Renken , and M. M. Lorist . 2014. “Brain Mechanisms Underlying the Effects of Aging on Different Aspects of Selective Attention.” NeuroImage 91: 52–62. 10.1016/j.neuroimage.2014.01.029. 24473095

[ejn70001-bib-0053] Green, P. J. , and B. W. Silverman . 1994. Nonparametric Regression and Generalized Linear Models: A Roughness Penalty Approach. London, UK: Chapman and Hall Ltd.

[ejn70001-bib-0054] Guevara, M. A. , E. I. C. Paniagua , M. H. González , et al. 2018. “EEG Activity During the Spatial Span Task in Young Men: Differences Between Short‐Term and Working Memory.” Brain Research 1683: 86–94. 29425909 10.1016/j.brainres.2018.02.004

[ejn70001-bib-0055] Güngen, C. , T. Ertan , E. Eker , R. Yaşar , and F. Engin . 2002. “Standardize Mini Mental Test'in Türk Toplumunda Hafif Demans Tanısında Geçerlik ve Güvenilirliği.” Türk Psikiyatri Dergisi 13: 273–281. 12794644

[ejn70001-bib-0056] Gutchess, A. , and A. Boduroglu . 2019. “Cultural Differences in Categorical Memory Errors Persist With Age.” Aging & Mental Health 23, no. 7: 851–854. 10.1080/13607863.2017.1421616. 29293028

[ejn70001-bib-0057] Hamasaki, A. , N. Akazawa , T. Yoshikawa , K. Myoenzono , K. Tagawa , and S. Maeda . 2018. “Age‐Related Declines in Executive Function and Cerebral Oxygenation Hemodynamics.” Tohoku Journal of Experimental Medicine 245, no. 4: 245–250. 10.1620/tjem.245.245. 30101827

[ejn70001-bib-0058] Hinault, T. , M. Mijalkov , J. B. Pereira , G. Volpe , A. Bakke , and S. M. Courtney . 2021. “Age‐Related Differences in Network Structure and Dynamic Synchrony of Cognitive Control.” NeuroImage 236: 118070. 10.1016/j.neuroimage.2021.118070. 33887473

[ejn70001-bib-0059] Holcomb, A. N. , C. F. Tagliabue , and V. Mazza . 2022. “Aging and Feature Binding in Visual Working Memory.” Frontiers in Psychology 13: 977565. 10.3389/fpsyg.2022.977565. 36275238 PMC9583905

[ejn70001-bib-0060] Hong, X. , Y. Liu , J. Sun , and S. Tong . 2016. “Age‐Related Differences in the Modulation of Small‐World Brain Networks During a Go/NoGo Task.” Frontiers in Aging Neuroscience 8: 100. 10.3389/fnagi.2016.00100. 27242512 PMC4869596

[ejn70001-bib-0061] Honkanen, R. , S. Rouhinen , S. H. Wang , J. M. Palva , and S. Palva . 2015. “Gamma Oscillations Underlie the Maintenance of Feature‐Specific Information and the Contents of Visual Working Memory.” Cerebral Cortex 25, no. 10: 3788–3801. 10.1093/cercor/bhu263. 25405942

[ejn70001-bib-0062] Hou, F. , C. Liu , Z. Yu , et al. 2018. “Age‐Related Alterations in Electroencephalography Connectivity and Network Topology During n‐Back Working Memory Task.” Frontiers in Human Neuroscience 12: 484. 10.3389/fnhum.2018.00484. 30574079 PMC6291464

[ejn70001-bib-0063] Iordan, A. D. , K. D. Moored , B. Katz , et al. 2021. “Age Differences in Functional Network Reconfiguration With Working Memory Training.” Human Brain Mapping 42, no. 6: 1888–1909. 10.1002/hbm.25337. 33534925 PMC7978135

[ejn70001-bib-0065] Javaid, H. , E. Kumarnsit , and S. Chatpun . 2022. “Age‐Related Alterations in EEG Network Connectivity in Healthy Aging.” Brain Sciences 12, no. 2: 218. 10.3390/brainsci12020218. 35203981 PMC8870284

[ejn70001-bib-0066] Johnson, E. L. , D. King‐Stephens , P. B. Weber , K. D. Laxer , J. J. Lin , and R. T. Knight . 2019. “Spectral Imprints of Working Memory for Everyday Associations in the Frontoparietal Network.” Frontiers in Systems Neuroscience 12: 65. 10.3389/fnsys.2018.00065. 30670953 PMC6333050

[ejn70001-bib-0067] Johnson, W. , R. H. Logie , and J. R. Brockmole . 2010. “Working Memory Tasks Differ in Factor Structure Across Age Cohorts: Implications for Dedifferentiation.” Intelligence 38, no. 5: 513–528.

[ejn70001-bib-0068] Kakkos, I. , G. N. Dimitrakopoulos , L. Gao , et al. 2019. “Mental Workload Drives Different Reorganizations of Functional Cortical Connectivity Between 2D and 3D Simulated Flight Experiments.” IEEE Transactions on Neural Systems and Rehabilitation Engineering 27, no. 9: 1704–1713. 10.1109/TNSRE.2019.2930082. 31329123

[ejn70001-bib-0069] Kaplan, E. , H. Goodglass , and S. Weintraub . 2001. Boston Naming Test, 2nd ed. Philadelphia, PA: Lippincott, Williams & Wilkins.

[ejn70001-bib-0070] Kitzbichler, M. G. , R. N. Henson , M. L. Smith , P. J. Nathan , and E. T. Bullmore . 2011. “Cognitive Effort Drives Workspace Configuration of Human Brain Functional Networks.” Journal of Neuroscience 31, no. 22: 8259–8270. 10.1523/JNEUROSCI.0440-11.2011. 21632947 PMC6622866

[ejn70001-bib-0071] Klimesch, W. 2012. “α‐Band Oscillations, Attention, and Controlled Access to Stored Information.” Trends in Cognitive Sciences 16, no. 12: 606–617. 10.1016/j.tics.2012.10.007. 23141428 PMC3507158

[ejn70001-bib-0072] Knyazev, G. G. , N. V. Volf , and L. V. Belousova . 2015. “Age‐Related Differences in Electroencephalogram Connectivity and Network Topology.” Neurobiology of Aging 36, no. 5: 1849–1859. 10.1016/j.neurobiolaging.2015.02.007. 25766772

[ejn70001-bib-0073] Kolskår, K. K. , D. Alnæs , T. Kaufmann , et al. 2018. “Key Brain Network Nodes Show Differential Cognitive Relevance and Developmental Trajectories During Childhood and Adolescence.” eNeuro 5, no. 4: ENEURO.0092‐18.2018. 10.1523/ENEURO.0092-18.2018. PMC6071203 30073200

[ejn70001-bib-0074] Lafer‐Sousa, R. , and B. R. Conway . 2013. “Parallel, Multi‐Stage Processing of Colors, Faces and Shapes in Macaque Inferior Temporal Cortex.” Nature Neuroscience 16, no. 12: 1870–1878. 24141314 10.1038/nn.3555 PMC3957328

[ejn70001-bib-1004] Langer, N. , A. Pedroni , and L. Jäncke . 2013. “The problem of Thresholding in Small‐World Network Analysis.” PLoS ONE 8, no. 1: e53199. 10.1371/journal.pone.0053199. 23301043 PMC3536769

[ejn70001-bib-0075] LaRocque, J. J. , J. A. Lewis‐Peacock , A. T. Drysdale , K. Oberauer , and B. R. Postle . 2013. “Decoding Attended Information in Short‐Term Memory: An EEG Study.” Journal of Cognitive Neuroscience 25, no. 1: 127–142. 10.1162/jocn_a_00305. 23198894 PMC3775605

[ejn70001-bib-0076] Latora, V. , and M. Marchiori . 2001. “Efficient Behavior of Small‐World Networks.” Physical Review Letters 87, no. 19: 198701. 10.1103/PhysRevLett.87.198701. 11690461

[ejn70001-bib-0077] Lau, T. M. , J. T. Gwin , K. G. McDowell , and D. P. Ferris . 2012. “Weighted Phase Lag Index Stability as an Artifact Resistant Measure to Detect Cognitive EEG Activity During Locomotion.” Journal of Neuroengineering and Rehabilitation 9: 47. 10.1186/1743-0003-9-47. 22828128 PMC3488562

[ejn70001-bib-0078] Lawhern, V. J. , A. J. Solon , N. R. Waytowich , S. M. Gordon , C. P. Hung , and B. J. Lance . 2018. “EEGNet: A Compact Convolutional Neural Network for EEG‐Based Brain‐Computer Interfaces.” Journal of Neural Engineering 15, no. 5: 056013. 10.1088/1741-2552/aace8c. 29932424

[ejn70001-bib-0079] Lee, H. , J. H. Jung , S. Chung , et al. 2023. “Graph Theoretical Analysis of Brain Structural Connectivity in Patients With Alcohol Dependence.” Experimental Neurobiology 32, no. 5: 362–369. 10.5607/en23026. 37927134 PMC10628861

[ejn70001-bib-0080] Leitgeb, E. P. , M. Šterk , T. Petrijan , P. Gradišnik , and M. Gosak . 2020. “The Brain as a Complex Network: Assessment of EEG‐Based Functional Connectivity Patterns in Patients With Childhood Absence Epilepsy.” Epileptic Disorders 22, no. 5: 519–530. 10.1684/epd.2020.1203. 33052105

[ejn70001-bib-0081] Lezak, M. D. , D. B. Howieson , E. D. Bigler , and D. Tranel . 2012. Neuropsychological Assessment. 5th ed. New York, NY: Oxford University Press.

[ejn70001-bib-0082] Li, G. , Y. Luo , Z. Zhang , et al. 2019. “Effects of Mental Fatigue on Small‐World Brain Functional Network Organization.” Neural Plasticity 2019: 1716074. 10.1155/2019/1716074. 31885535 PMC6918937

[ejn70001-bib-0083] Liang, X. , Q. Zou , Y. He , and Y. Yang . 2016. “Topologically Reorganized Connectivity Architecture of Default‐Mode, Executive‐Control, and Salience Networks Across Working Memory Task Loads.” Cerebral Cortex 26: 1501–1511. 25596593 10.1093/cercor/bhu316 PMC4785946

[ejn70001-bib-0084] Liang, Y. , Y. Pertzov , J. M. Nicholas , et al. 2016. “Visual Short‐Term Memory Binding Deficit in Familial Alzheimer's Disease.” Cortex 78: 150–164. 10.1016/j.cortex.2016.01.015. 27085491 PMC4865502

[ejn70001-bib-0085] López, M. E. , S. Aurtenetxe , E. Pereda , et al. 2014. “Cognitive Reserve Is Associated With the Functional Organization of the Brain in Healthy Aging: A MEG Study.” Frontiers in Aging Neuroscience 6: 125. 10.3389/fnagi.2014.00125. 24982632 PMC4056015

[ejn70001-bib-0086] Luck, S. J. , and E. K. Vogel . 1997. “The Capacity of Visual Working Memory for Features and Conjunctions.” Nature 390, no. 6657: 279–281. 10.1038/36846. 9384378

[ejn70001-bib-0087] Ma, J. , J. Zhang , Y. Lin , and Z. Dai . 2021. “Cost‐Efficiency Trade‐Offs of the Human Brain Network Revealed by a Multiobjective Evolutionary Algorithm.” NeuroImage 236: 118040. 10.1016/j.neuroimage.2021.118040. 33852939

[ejn70001-bib-0088] Mamat, M. , Z. Wang , L. Jin , K. He , L. Li , and Y. Chen . 2024. “Beyond Nodes and Edges: A Bibliometric Analysis on Graph Theory and Neuroimaging Modalities.” Frontiers in Neuroscience 18: 1373264. 10.3389/fnins.2024.1373264. 38716254 PMC11074400

[ejn70001-bib-0089] Maslov, S. , and K. Sneppen . 2002. “Specificity and Stability in Topology of Protein Networks.” Science 296, no. 5569: 910–913. 10.1126/science.1065103. 11988575

[ejn70001-bib-0090] McCullagh, P. , and J. A. Nelder . 1989. Generalized Linear Models. 2nd ed. New York, NY: Chapman and Hall, Inc.

[ejn70001-bib-0091] Medaglia, J. D. , W. Huang , E. A. Karuza , et al. 2018. “Functional Alignment With Anatomical Networks Is Associated With Cognitive Flexibility.” Nature Human Behaviour 2, no. 2: 156–164. 10.1038/s41562-017-0260-9. PMC6258039 30498789

[ejn70001-bib-0092] Meunier, D. , R. Lambiotte , and E. T. Bullmore . 2010. “Modular and Hierarchically Modular Organization of Brain Networks.” Frontiers in Neuroscience 4: 200. 10.3389/fnins.2010.00200. 21151783 PMC3000003

[ejn70001-bib-0093] Meunier, D. , E. A. Stamatakis , and L. K. Tyler . 2014. “Age‐Related Functional Reorganization, Structural Changes, and Preserved Cognition.” Neurobiology of Aging 35, no. 1: 42–54. 10.1016/j.neurobiolaging.2013.07.003. 23942392

[ejn70001-bib-0094] Mheich, A. , F. Wendling , and M. Hassan . 2020. “Brain Network Similarity: Methods and Applications.” Network Neuroscience 4, no. 3: 507–527. 10.1162/netn_a_00133. 32885113 PMC7462433

[ejn70001-bib-0095] Mizuhara, H. , and Y. Yamaguchi . 2007. “Human Cortical Circuits for Central Executive Function Emerge by Theta Phase Synchronization.” NeuroImage 36, no. 1: 232–244. 10.1016/j.neuroimage.2007.02.026. 17433880

[ejn70001-bib-0096] Murman, D. L. 2015. “The Impact of Age on Cognition.” Seminars in Hearing 36, no. 3: 111–121. 10.1055/s-0035-1555115. 27516712 PMC4906299

[ejn70001-bib-0097] Niso, G. , R. Bruña , E. Pereda , et al. 2013. “HERMES: Towards an Integrated Toolbox to Characterize Functional and Effective Brain Connectivity.” Neuroinformatics 11, no. 4: 405–434. 10.1007/s12021-013-9186-1. 23812847

[ejn70001-bib-0098] Oosterhuis, E. J. , K. Slade , P. J. C. May , and H. E. Nuttall . 2023. “Toward an Understanding of Healthy Cognitive Aging: The Importance of Lifestyle in Cognitive Reserve and the Scaffolding Theory of Aging and Cognition.” Journals of Gerontology. Series B, Psychological Sciences and Social Sciences 78, no. 5: 777–788. 10.1093/geronb/gbac197. 36546399 PMC10174283

[ejn70001-bib-0099] Ortiz, E. , K. Stingl , J. Münssinger , C. Braun , H. Preissl , and P. Belardinelli . 2012. “Weighted Phase Lag Index and Graph Analysis: Preliminary Investigation of Functional Connectivity During Resting State in Children.” Computational and Mathematical Methods in Medicine 2012: 186353. 10.1155/2012/186353. 23049617 PMC3462418

[ejn70001-bib-0100] Osipova, D. , A. Takashima , R. Oostenveld , G. Fernández , E. Maris , and O. Jensen . 2006. “Theta and Gamma Oscillations Predict Encoding and Retrieval of Declarative Memory.” Journal of Neuroscience 26, no. 28: 7523–7531. 10.1523/JNEUROSCI.1948-06.2006. 16837600 PMC6674196

[ejn70001-bib-0101] Palva, J. M. , S. Monto , S. Kulashekhar , and S. Palva . 2010. “Neuronal Synchrony Reveals Working Memory Networks and Predicts Individual Memory Capacity.” Proceedings of the National Academy of Sciences of the United States of America 107, no. 16: 7580–7585. 10.1073/pnas.0913113107. 20368447 PMC2867688

[ejn70001-bib-0102] Parra, M. A. , S. Abrahams , K. Fabi , R. Logie , S. Luzzi , and S. Della Sala . 2009. “Short‐Term Memory Binding Deficits in Alzheimer's Disease.” Brain 132, no. Pt 4: 1057–1066. 10.1093/brain/awp036. 19293236

[ejn70001-bib-0103] Parra, M. A. , S. Abrahams , R. H. Logie , and S. Della Sala . 2010. “Visual Short‐Term Memory Binding in Alzheimer's Disease and Depression.” Journal of Neurology 257, no. 7: 1160–1169. 10.1007/s00415-010-5484-9. 20162428

[ejn70001-bib-0104] Parra, M. A. , S. Abrahams , R. H. Logie , L. G. Méndez , F. Lopera , and S. Della Sala . 2010. “Visual Short‐Term Memory Binding Deficits in Familial Alzheimer's Disease.” Brain 133, no. 9: 2702–2713. 10.1093/brain/awq148. 20624814

[ejn70001-bib-0105] Parra, M. A. , S. Abrahams , R. H. Logie , and S. D. Sala . 2009. “Age and Binding Within‐Dimension Features in Visual Short‐Term Memory.” Neuroscience Letters 449: 1–5. 10.1016/j.neulet.2008.10.069. 18977410

[ejn70001-bib-1005] Parra, M. A. , E. Mikulan , N. Trujillo , et al. 2017. “Brain Information Sharing During Visual Short‐Term Memory Binding Yields a Memory Biomarker for Familial Alzheimer's Disease.” Current Alzheimer Research 14, no. 12: 1335–1347. 10.2174/1567205014666170614163316. 28641509

[ejn70001-bib-0106] Pavisic, I. M. , A. Suarez‐Gonzalez , and Y. Pertzov . 2020. “Translating Visual Short‐Term Memory Binding Tasks to Clinical Practice: From Theory to Practice.” Frontiers in Neurology 11: 458. 10.3389/fneur.2020.00458. 32587567 PMC7297911

[ejn70001-bib-0107] Pedersen, R. , L. Geerligs , M. Andersson , et al. 2021. “When Functional Blurring Becomes Deleterious: Reduced System Segregation Is Associated With Less White Matter Integrity and Cognitive Decline in Aging.” NeuroImage 242: 118449. 10.1016/j.neuroimage.2021.118449. 34358662

[ejn70001-bib-0108] Phillips, L. H. , and P. Andrés . 2010. “The Cognitive Neuroscience of Aging: New Findings on Compensation and Connectivity.” Cortex 46, no. 4: 421–424. 10.1016/j.cortex.2010.01.005. 20132931

[ejn70001-bib-0109] Pievani, M. , N. Filippini , M. P. van den Heuvel , S. F. Cappa , and G. B. Frisoni . 2014. “Brain Connectivity in Neurodegenerative Diseases—From Phenotype to Proteinopathy.” Nature Reviews. Neurology 10, no. 11: 620–633. 10.1038/nrneurol.2014.178. 25287597

[ejn70001-bib-0110] Pijnenburg, Y. A. , Y. van der Made , A. M. van Cappellen van Walsum , D. L. Knol , P. Scheltens , and C. J. Stam . 2004. “EEG Synchronization Likelihood in Mild Cognitive Impairment and Alzheimer's Disease During a Working Memory Task.” Clinical Neurophysiology 115, no. 6: 1332–1339. 10.1016/j.clinph.2003.12.029. 15134700

[ejn70001-bib-0111] Rakesh, D. , K. B. Fernando , and L. S. Mansour . 2020. “Functional Dedifferentiation of the Brain During Healthy Aging.” Journal of Neurophysiology 123, no. 4: 1279–1282. 10.1152/jn.00039.2020. 32130084

[ejn70001-bib-1006] Ren, S. , J. Li , F. Taya , J. deSouza , N. V. Thakor , and A. Bezerianos . 2017. “Dynamic Functional Segregation and Integration in Human Brain Network During Complex Tasks.” IEEE Transactions on Neural Systems and Rehabilitation Engineering 25, no. 6: 547–556. 10.1109/TNSRE.2016.2597961. 28113670

[ejn70001-bib-0112] Rhodes, S. , M. A. Parra , and R. H. Logie . 2016. “Ageing and Feature Binding in Visual Working Memory: The Role of Presentation Time.” Quarterly Journal of Experimental Psychology 69: 654–668. 10.1080/17470218.2015.1038571. 25993530

[ejn70001-bib-0113] Rose, N. S. , J. Myerson , M. S. Sommers , and S. Hale . 2009. “Are There Age Differences in the Executive Component of Working Memory? Evidence From Domain‐General Interference Effects.” Aging, Neuropsychology, and Cognition 16, no. 6: 633–653. 10.1080/13825580902825238. PMC2823116 19401863

[ejn70001-bib-1007] Rubinov, M. , and O. Sporns . 2010. “Complex Network Measures of Brain Connectivity: Uses and Interpretations.” NeuroImage 52, no. 3: 1059–1069. 10.1016/j.neuroimage.2009.10.003. 19819337

[ejn70001-bib-0114] Salthouse, T. A. 2010a. Major Issues in Cognitive Aging. New York: Oxford University Press.

[ejn70001-bib-0115] Salthouse, T. A. 2010b. “Selective Review of Cognitive Aging.” Journal of the International Neuropsychological Society 16, no. 5: 754–760. 10.1017/S1355617710000706. 20673381 PMC3637655

[ejn70001-bib-1008] Sander, M. C. , U. Lindenberger , and M. Werkle‐Bergner . 2012. “Lifespan Age Differences in Working Memory: A Two‐Component Framework.” Neuroscience and Biobehavioral Reviews 36, no. 9: 2007–2033. 10.1016/j.neubiorev.2012.06.004. 22771333

[ejn70001-bib-0116] Sander, M. C. , M. Werkle‐Bergner , and U. Lindenberger . 2011. “Contralateral Delay Activity Reveals Life‐Span Age Differences in Top‐Down Modulation of Working Memory Contents.” Cerebral Cortex 21, no. 12: 2809–2819. 10.1093/cercor/bhr076. 21527784

[ejn70001-bib-0117] Sauseng, P. , J. Hoppe , W. Klimesch , C. Gerloff , and F. C. Hummel . 2007. “Dissociation of Sustained Attention From Central Executive Functions: Local Activity and Interregional Connectivity in the Theta Range.” European Journal of Neuroscience 25, no. 2: 587–593. 10.1111/j.1460-9568.2006.05286.x. 17284201

[ejn70001-bib-0118] Sauseng, P. , W. Klimesch , M. Doppelmayr , T. Pecherstorfer , R. Freunberger , and S. Hanslmayr . 2005. “EEG Alpha Synchronization and Functional Coupling During Top‐Down Processing in a Working Memory Task.” Human Brain Mapping 26, no. 2: 148–155. 10.1002/hbm.20150. 15929084 PMC6871735

[ejn70001-bib-0119] Sauseng, P. , W. Klimesch , K. F. Heise , et al. 2009. “Brain Oscillatory Substrates of Visual Short‐Term Memory Capacity.” Current Biology 19, no. 21: 1846–1852. 10.1016/j.cub.2009.08.062. 19913428

[ejn70001-bib-0120] Shafritz, K. M. , J. C. Gore , and R. Marois . 2002. “The Role of the Parietal Cortex in Visual Feature Binding.” Proceedings of the National Academy of Sciences 99: 10917–10922. 10.1073/pnas.152694799. PMC125073 12149449

[ejn70001-bib-0121] Shine, J. M. 2019. “Neuromodulatory Influences on Integration and Segregation in the Brain.” Trends in Cognitive Sciences 23, no. 7: 572–583. 10.1016/j.tics.2019.04.002. 31076192

[ejn70001-bib-0122] Shine, J. M. , P. G. Bissett , P. T. Bell , et al. 2016. “The Dynamics of Functional Brain Networks: Integrated Network States During Cognitive Task Performance.” Neuron 92, no. 2: 544–554. 10.1016/j.neuron.2016.09.018. 27693256 PMC5073034

[ejn70001-bib-0123] Smit, D. J. , M. Boersma , C. E. van Beijsterveldt , et al. 2010. “Endophenotypes in a Dynamically Connected Brain.” Behavior Genetics 40, no. 2: 167–177. 10.1007/s10519-009-9330-8. 20111993 PMC2829652

[ejn70001-bib-0125] Sporns, O. 2007. “Brain Connectivity.” Scholarpedia 2, no. 10: 4695.

[ejn70001-bib-0126] Sporns, O. 2013. “The Human Connectome: Origins and Challenges.” NeuroImage 80: 53–61. 10.1016/j.neuroimage.2013.03.023. 23528922

[ejn70001-bib-0127] Sporns, O. , and R. F. Betzel . 2016. “Modular Brain Networks.” Annual Review of Psychology 67: 613–640. 10.1146/annurev-psych-122414-033634. PMC4782188 26393868

[ejn70001-bib-0128] Sporns, O. , C. J. Honey , and R. Kötter . 2007. “Identification and Classification of Hubs in Brain Networks.” PLoS ONE 2, no. 10: e1049. 10.1371/journal.pone.0001049. 17940613 PMC2013941

[ejn70001-bib-0129] Sporns, O. , and J. D. Zwi . 2004. “The Small World of the Cerebral Cortex.” Neuroinformatics 2, no. 2: 145–162. 10.1385/NI:2:2:145. 15319512

[ejn70001-bib-0130] Spreen, O. , and E. Strauss . 1998. A Compendium of Psychological Tests. New York: Oxford University Press.

[ejn70001-bib-0131] Stam, C. J. , and B. W. van Dijk . 2002. “Synchronization Likelihood: An Unbiased Measure of Generalized Synchronization in Multivariate Data Sets.” Physica D: Nonlinear Phenomena 163: 236–251.

[ejn70001-bib-0132] Stanley, M. L. , D. Dagenbach , R. G. Lyday , J. H. Burdette , and P. J. Laurienti . 2014. “Changes in Global and Regional Modularity Associated With Increasing Working Memory Load.” Frontiers in Human Neuroscience 8: 954. 25520639 10.3389/fnhum.2014.00954 PMC4249452

[ejn70001-bib-0134] Stern, Y. 2009. “Cognitive Reserve.” Neuropsychologia 47, no. 10: 2015–2028. 10.1016/j.neuropsychologia.2009.03.004. 19467352 PMC2739591

[ejn70001-bib-0135] Stevens, A. A. , S. C. Tappon , A. Garg , and D. A. Fair . 2012. “Functional Brain Network Modularity Captures Inter‐ and Intra‐Individual Variation in Working Memory Capacity.” PLoS ONE 7, no. 1: e30468. 10.1371/journal.pone.0030468. 22276205 PMC3262818

[ejn70001-bib-0136] Tanör, Ö. Ö. 2011. “Öktem Sözel Bellek Süreçleri Testi. (Öktem‐SBST) el Kitabı.”

[ejn70001-bib-0137] Tegin, B. 1980. “Turkish Version of Beck Depression Inventory.” Unpublished Doctorate Thesis.

[ejn70001-bib-0138] Teng, C. , Y. Cheng , C. Wang , Y. Ren , W. Xu , and J. Xu . 2018. “Aging‐Related Changes of EEG Synchronization During a Visual Working Memory Task.” Cognitive Neurodynamics 12: 561–568. 30483364 10.1007/s11571-018-9500-6 PMC6233325

[ejn70001-bib-0139] Tobe, M. , S. Nobukawa , K. Mizukami , et al. 2023. “Hub Structure in Functional Network of EEG Signals Supporting High Cognitive Functions in Older Individuals.” Frontiers in Aging Neuroscience 15: 1130428. 10.3389/fnagi.2023.1130428. 37139091 PMC10149684

[ejn70001-bib-0140] Todd, J. J. , and R. Marois . 2004. “Capacity Limit of Visual Short‐Term Memory in Human Posterior Parietal Cortex.” Nature 428, no. 6984: 751–754. 10.1038/nature02466. 15085133

[ejn70001-bib-0141] Toppi, J. , L. Astolfi , M. Risetti , et al. 2018. “Different Topological Properties of EEG‐Derived Networks Describe Working Memory Phases as Revealed by Graph Theoretical Analysis.” Frontiers in Human Neuroscience 11: 637. 10.3389/fnhum.2017.00637. 29379425 PMC5770976

[ejn70001-bib-0142] Treisman, A. , and W. Zhang . 2006. “Location and Binding in Visual Working Memory.” Memory & Cognition 34, no. 8: 1704–1719. 10.3758/bf03195932. 17489296 PMC1868390

[ejn70001-bib-0143] Tseng, P. , Y. T. Chang , C. F. Chang , W. K. Liang , and C. H. Juan . 2016. “The Critical Role of Phase Difference in Gamma Oscillation Within the Temporoparietal Network for Binding Visual Working Memory.” Scientific Reports 6: 32138. 10.1038/srep32138. 27573864 PMC5004173

[ejn70001-bib-0144] Tumaç, A. 1997. Normal Deneklerde Frontal Hasarlara Duyarlı Bazı Testlerde Performansa yaş ve Eğitimin Etkisi.” Yayınlanmamış Psikoloji Yüksek Lisans Tezi. İstanbul Üniversitesi, Sosyal Bilimler Enstitüsü.

[ejn70001-bib-0145] Unsworth, N. , K. Fukuda , E. Awh , and E. K. Vogel . 2014. “Working Memory and Fluid Intelligence: Capacity, Attention Control, and Secondary Memory Retrieval.” Cognitive Psychology 71: 1–26. 10.1016/j.cogpsych.2014.01.003. 24531497 PMC4484859

[ejn70001-bib-0146] van den Heuvel, M. P. , R. C. Mandl , C. J. Stam , R. S. Kahn , and H. E. Hulshoff Pol . 2010. “Aberrant Frontal and Temporal Complex Network Structure in Schizophrenia: A Graph Theoretical Analysis.” Journal of Neuroscience 30, no. 47: 15915–15926. 10.1523/JNEUROSCI.2874-10.2010. 21106830 PMC6633761

[ejn70001-bib-1009] van den Heuvel, M. P. , and O. Sporns . 2013. “Network Hubs in the Human Brain.” Trends in Cognitive Sciences 17, no. 12: 683–696. 10.1016/j.tics.2013.09.012. 24231140

[ejn70001-bib-0147] Vecchio, F. , F. Miraglia , P. Bramanti , and P. M. Rossini . 2014. “Human Brain Networks in Physiological Aging: A Graph Theoretical Analysis of Cortical Connectivity From EEG Data.” Journal of Alzheimer's Disease 41, no. 4: 1239–1249. 10.3233/JAD-140090. 24820018

[ejn70001-bib-0148] Vinck, M. , R. Oostenveld , M. van Wingerden , F. Battaglia , and C. M. Pennartz . 2011. “An Improved Index of Phase‐Synchronization for Electrophysiological Data in the Presence of Volume‐Conduction, Noise and Sample‐Size Bias.” NeuroImage 55, no. 4: 1548–1565. 10.1016/j.neuroimage.2011.01.055. 21276857

[ejn70001-bib-0149] Vogel, E. K. , and M. G. Machizawa . 2004. “Neural Activity Predicts Individual Differences in Visual Working Memory Capacity.” Nature 428, no. 6984: 748–751. 10.1038/nature02447. 15085132

[ejn70001-bib-0150] Wang, L. , W. Wang , T. Yan , et al. 2017. “Beta‐Band Functional Connectivity Influences Audiovisual Integration in Older Age: An EEG Study.” Frontiers in Aging Neuroscience 9: 239. 10.3389/fnagi.2017.00239. 28824411 PMC5545595

[ejn70001-bib-0151] Wang, R. , M. Liu , X. Cheng , Y. Wu , A. Hildebrandt , and C. Zhou . 2021. “Segregation, Integration, and Balance of Large‐Scale Resting Brain Networks Configure Different Cognitive Abilities.” Proceedings of the National Academy of Sciences of the United States of America 118, no. 23: e2022288118. 10.1073/pnas.2022288118. 34074762 PMC8201916

[ejn70001-bib-0152] Watts, D. J. , and S. H. Strogatz . 1998. “Collective Dynamics of ‘Small‐World’ Networks.” Nature 393, no. 6684: 440–442. 10.1038/30918. 9623998

[ejn70001-bib-0153] Wechsler, D. 1987. Wechsler Memory Scale‐Revised. San Antonio, TX: Psychological Corporation.

[ejn70001-bib-0154] Wechsler, D. 1997a. Wechsler Memory Scale—Third Edition Manual. San Antonio, TX: Psychological Corporation.

[ejn70001-bib-0155] Wechsler, D. 1997b. WAIS‐III/WMS‐III Technical Manual. San Antonio, TX: Psychological Corporation.

[ejn70001-bib-0156] Wiegand, I. , M. J. Lauritzen , M. Osler , et al. 2018. “EEG Correlates of Visual Short‐Term Memory in Older Age Vary With Adult Lifespan Cognitive Development.” Neurobiology of Aging 62: 210–220. 10.1016/j.neurobiolaging.2017.10.018. 29175710

[ejn70001-bib-0157] Wiegand, I. , T. Töllner , M. Dyrholm , H. J. Müller , C. Bundesen , and K. Finke . 2014. “Neural Correlates of Age‐Related Decline and Compensation in Visual Attention Capacity.” Neurobiology of Aging 35, no. 9: 2161–2173. 10.1016/j.neurobiolaging.2014.02.023. 24684790

[ejn70001-bib-0158] Wijeakumar, S. , V. A. Magnotta , and J. P. Spencer . 2017. “Modulating Perceptual Complexity and Load Reveals Degradation of the Visual Working Memory Network in Ageing.” NeuroImage 157: 464–475. 10.1016/j.neuroimage.2017.06.019. 28627364

[ejn70001-bib-0159] Wischnewski, M. , T. A. Berger , A. Opitz , and I. Alekseichuk . 2024. “Causal Functional Maps of Brain Rhythms in Working Memory.” Proceedings of the National Academy of Sciences of the United States of America 121, no. 14: e2318528121. 10.1073/pnas.2318528121. 38536752 PMC10998564

[ejn70001-bib-0160] Wodeyar, A. , and R. Srinivasan . 2018. “Network Structure During Encoding Predicts Working Memory Performance.” BioRxiv. 10.1101/409615.

[ejn70001-bib-0161] Wolff, M. J. , J. Ding , N. E. Myers , and M. G. Stokes . 2015. “Revealing Hidden States in Visual Working Memory Using Electroencephalography.” Frontiers in Systems Neuroscience 9: 123. 10.3389/fnsys.2015.00123. 26388748 PMC4558475

[ejn70001-bib-0162] Wood, S. N. 2017. Generalized Additive Models: An Introduction With R. 2nd ed. Boca Raton, FL: Chapman and Hall/CRC. 10.1201/9781315370279.

[ejn70001-bib-0163] Xiang, N. , Q. Wang , and M. You . 2023. “Estimation and Update of Betweenness Centrality With Progressive Algorithm and Shortest Paths Approximation.” Scientific Reports 13, no. 1: 17110. 10.1038/s41598-023-44392-0. 37816806 PMC10564764

[ejn70001-bib-0164] Xu, T. , J. Huang , Z. Pei , et al. 2023. “The Effect of Multiple Factors on Working Memory Capacities: Aging, Task Difficulty, and Training.” IEEE Transactions on Biomedical Engineering 70, no. 6: 1967–1978. 10.1109/TBME.2022.3232849. 37015624

[ejn70001-bib-0165] Yoshinaga, K. , M. Matsuhashi , T. Mima , et al. 2020. “Comparison of Phase Synchronization Measures for Identifying Stimulus‐Induced Functional Connectivity in Human Magnetoencephalographic and Simulated Data.” Frontiers in Neuroscience 14: 648. 10.3389/fnins.2020.00648. 32636735 PMC7318889

[ejn70001-bib-0166] Zamani Esfahlani, F. , Y. Jo , M. G. Puxeddu , et al. 2021. “Modularity Maximization as a Flexible and Generic Framework for Brain Network Exploratory Analysis.” NeuroImage 244: 118607. 10.1016/j.neuroimage.2021.118607. 34607022

[ejn70001-bib-0167] Zanto, T. P. , and A. Gazzaley . 2009. “Neural Suppression of Irrelevant Information Underlies Optimal Working Memory Performance.” Journal of Neuroscience 29, no. 10: 3059–3066. 10.1523/JNEUROSCI.4621-08.2009. 19279242 PMC2704557

[ejn70001-bib-0168] Zarahn, E. , B. Rakitin , D. Abela , J. Flynn , and Y. Stern . 2007. “Age‐Related Changes in Brain Activation During a Delayed Item Recognition Task.” Neurobiology of Aging 28, no. 5: 784–798. 10.1016/j.neurobiolaging.2006.03.002. 16621168

[ejn70001-bib-0169] Zhang, S. , Y. Zhang , Y. Sun , N. Thakor , and A. Bezerianos . 2017. “Graph Theoretical Analysis of EEG Functional Network During Multi‐Workload Flight Simulation Experiment in Virtual Reality Environment.” Annual International Conference of the IEEE Engineering in Medicine and Biology Society 2017: 3957–3960. 10.1109/EMBC.2017.8037722. 29060763

[ejn70001-bib-1010] Zippo, A. G. , P. A. Della Rosa , I. Castiglioni , and G. E. M. Biella . 2018. “Alternating Dynamics of Segregation and Integration in Human EEG Functional Networks During Working‐Memory Task.” Neuroscience 371: 191–206. 10.1016/j.neuroscience.2017.12.004. 29246785

